# Mitochondrial ultrastructural pathology in diabetic cardiomyopathy: integrated analysis *via* scanning electron microscopy and 3D visualization imaging

**DOI:** 10.1186/s12933-025-02884-5

**Published:** 2025-08-13

**Authors:** Bo Wang, Linghao Dai, Haowei Liang, Jiayu He, Jiayi Zhou, Yang Guan, Hui Wang

**Affiliations:** 1 Zhejiang Chinese Medical University, Hangzhou, China; 2 Jinhua Academy, Zhejiang Chinese Medical University, Jinhua, China; 3 Fenghua District Traditional Chinese Medicine Hospital of Ningbo, Ningbo, China

**Keywords:** Diabetic cardiomyopathy, Mitochondria, Ultrastructure, Energy metabolism, Scanning electron microscopy, 3D visualization imaging

## Abstract

**Background:**

Mitochondrial dysfunction plays a pivotal role in the onset and progression of diabetic cardiomyopathy (DCM). It is hypothesized that ultrastructural mitochondrial abnormalities, molecular dynamics imbalance, and bioenergetic impairments collectively contribute significantly to cardiac dysfunction. Consequently, investigating mitochondrial ultrastructural changes and metabolic disturbances is crucial for elucidating the mechanistic underpinnings of DCM.

**Objective:**

This study aims to comprehensively characterize alterations in mitochondrial ultrastructure and energy metabolism in DCM and examine the interplay between these two factors.

**Methods:**

High glucose-treated H9c2 cardiomyocytes and DCM model mice were analyzed via scanning electron microscopy (SEM) and 3D imaging. Three-dimensional morphometric parameters—such as Length3D, Thickness3D, Width3D, Area3D, Volume3D, Anisotropy, Flatness, and Elongation—were quantified to evaluate mitochondrial architecture. At the two-dimensional (2D) level, mitochondria-associated membrane (MAM) parameters were analyzed. Further, detailed statistical analysis was conducted on mitochondrial cristae, including cristae scores, count, width, gap size, and junction widths in myocardial tissues. Mitochondrial dynamics and autophagy-related protein expression (Mfn1, Mfn2, Opa1, p-Drp1(ser616), PINK1, Parkin1) in myocardial tissues were assessed by Western blot. Mitochondrial bioenergetics were measured by ATP content, membrane potential, mtDNA copy number, SOD levels, mitochondrial Ca^2+^ levels, and oxidative phosphorylation (OXPHOS) activity across Complexes I–V in myocardial tissue. Additionally, the oxygen consumption rate (OCR) of viable H9c2 cells was measured using the O2k system.

**Results:**

3D reconstruction revealed key myocardial ultrastructures—including T-tubules, sarcoplasmic reticulum (SR), intercalated discs, and mitochondria—and demonstrated significant differences in mitochondrial morphology and 3D morphometric parameters across subpopulations. Under high glucose (HG) conditions, in vitro analysis showed a reduction in mitochondrial Length3D and Anisotropy in H9c2 cells, accompanied by increases in Thickness3D, Width3D, Flatness, and Elongation. HG exposure also led to an increase in the length of MAM contact sites and the MAM-to-mitochondria perimeter ratio. In vivo, the DCM group exhibited decreased 2D morphometric parameters (length, width, area, perimeter, and shape AP), as well as reductions in 3D measurements (Thickness3D, Width3D, and Volume3D) compared to controls. No significant differences were observed in Length3D, Area3D, Anisotropy, Flatness, and Elongation between groups. 3D surface analysis revealed rough mitochondrial surfaces in the DCM group, while controls displayed smooth surfaces. Control mitochondria exhibited well-aligned, well-defined cristae, whereas DCM mitochondria showed cristae dissolution, disorganized arrangements, and vacuolization within the cristae. The DCM group also had increased cristae junction width and spacing. Additionally, megamitochondria were observed in DCM samples. The DCM group showed a significant increase in MAM contact site length and MAM-to-mitochondria perimeter ratio in myocardial tissue. Molecular analysis revealed decreased expression of fusion proteins (Mfn1, Opa1) and increased levels of p-Drp1(ser616), alongside elevated autophagy markers (PINK1, Parkin1). Bioenergetic dysfunction was evident through decreased ATP production, mitochondrial membrane potential collapse (ΔΨm), reduced mtDNA copy number, decreased SOD levels, impaired activities of complexes I/III/IV/V, and diminished basal/maximal respiration, ATP-linked respiration, and spare respiratory capacity. Conversely, mitochondrial Ca^2+^ levels were elevated in the DCM group, along with increased proton leakage in H9c2 cells.

**Conclusions:**

This study establishes a comprehensive framework linking “3D morphology–molecular regulation–metabolic dysfunction,” highlighting the synergistic interplay between mitochondrial ultrastructural remodeling and bioenergetic failure as key drivers of DCM progression. These findings provide valuable insights into DCM pathogenesis and suggest potential therapeutic targets.

**Supplementary Information:**

The online version contains supplementary material available at 10.1186/s12933-025-02884-5.

## Background

Diabetic cardiomyopathy (DCM) is a diabetes-induced myocardial disorder marked by both structural and functional cardiac abnormalities [1]. The pathogenesis of DCM is multifactorial, involving myocardial metabolic dysregulation, impaired cardiac insulin signaling, oxidative stress, chronic inflammation, mitochondrial dysfunction, and endoplasmic reticulum (ER) stress [2, 3]. Among these mechanisms, mitochondrial dysfunction is central to the initiation and progression of DCM. Cardiomyocytes have sustained high energy demands to support contractile activity, with mitochondria serving as the obligate ATP source essential for maintaining cardiac homeostasis. Mitochondrial dysfunction disrupts ATP synthesis, resulting in bioenergetic insufficiency and subsequent cardiomyocyte dysfunction. Thus, preserving mitochondrial integrity and function is critical for sustaining myocardial performance [4, 5]. Evidence suggests that environmental stressors, such as hyperglycemia and hyperlipidemia, induce mitochondrial ultrastructural disorganization, disturbing bioenergetic function and compromising physiological homeostasis. These findings highlight the significance of mitochondrial pathology in DCM research. Ultrastructural pathology studies provide insight into DCM etiology, pathogenesis, and the pathological alterations within cardiomyocytes and mitochondria. Conventional transmission electron microscopy (TEM) has been used for ultrastructural analysis; however, its reliance on two-dimensional (2D) sectional observations often fails to capture the spatial and volumetric complexities of the myocardium. Recent advancements in scanning electron microscopy (SEM) resolution and sample preparation techniques now allow for three-dimensional (3D) surface rendering and volumetric reconstruction *via* serial section imaging, positioning SEM as a pivotal tool in volume electron microscopy (vEM). Recent applications of vEM have revealed detailed ultrastructural features in various biological systems, including cellular, insect, and mouse tissues [6–8]. In cardiac research, Pinali et al. employed serial block-face SEM (SBF-SEM) to decode the 3D spatial network between T-tubules and the sarcoplasmic reticulum (SR) in mouse myocardium [9]. Beikoghli Kalkhoran characterized mitochondrial-SR junctional complexes in mitofusin-2 (MFN2)-deficient models [10]. Despite these advances, systematic investigations into 3D myocardial ultrastructure in pathological conditions, particularly regarding DCM-associated mitochondrial and tissue-level 3D remodeling, remain scarce.

Given the significant ultrastructural changes observed in DCM and the unmatched analytical power of 3D visualization technologies, this study integrates SEM-based surface imaging with serial section reconstruction to investigate myocardial ultrastructural alterations, with a specific focus on mitochondria, during DCM progression. Concurrent evaluations of mitochondrial bioenergetic capacity further elucidate the pathophysiological relationship between structural remodeling and metabolic dysfunction. By establishing a multidimensional “3D ultrastructure–bioenergetics” framework, this work not only advances the understanding of DCM from a 3D ultrastructural perspective but also provides novel mechanistic insights into its pathological progression.

## Methods​

### ​Establishment and validation of a mouse DCM model​

Six-week-old C57BL/6 mice (purchased from Zhejiang Chinese Medical University, Animal Ethics Approval Number: 20210419-01) underwent a 2-week acclimatization period before fasting for 24 h (with free access to water) and were then randomly divided into two groups: the control group (*n* = 10), which was fed a standard chow diet, and the experimental group, which received a high-fat diet (HFD, 60% fat by weight). After 6 weeks of dietary intervention, mice in the HFD group were intraperitoneally injected with streptozotocin (STZ, 100 mg/kg) for 3 consecutive days. Seven days after the final injection, mice were fasted for 12 h, and fasting blood glucose (FBG) levels were measured. Mice with FBG ≥ 16.6 mmol/L were considered to have successfully developed diabetes. After an additional 8 weeks of feeding, cardiac function was assessed using a small animal ultrasound imaging system (VisualSonics Vevo 3100). Anesthesia was induced with 3% isoflurane and maintained with 1% isoflurane during the ultrasound examination. Functional parameters, including left ventricular ejection fraction (LVEF) and left ventricular fractional shortening (LVFS), were recorded. Mice showing significantly reduced LVEF and LVFS compared to the control group were classified as the DCM model group.

Cardiac tissues were fixed with 4% paraformaldehyde, followed by graded alcohol dehydration (70–100%), paraffin embedding, and section preparation. Hematoxylin-eosin (H&E) staining was performed to observe the overall myocardial structure; Sirius Red staining was used to assess fibrotic deposition; and cell apoptosis was detected using a TUNEL staining kit (Beyotime, C1090). Additionally, OCT-embedded frozen sections were prepared for oil red O staining to visualize lipid accumulation and for reactive oxygen species (ROS) fluorescence staining (Beyotime, S0063). The cross-sectional area of cardiomyocytes in H&E-stained sections was measured using Amira software, while the proportion of Sirius Red-positive areas, oil red O-positive areas, and the number of TUNEL/ROS-positive cells were quantified *via* ImageJ software.

### ​High-glucose-induced cardiomyocyte model​

H9c2 cardiomyoblasts (obtained from the Chinese Academy of Sciences Cell Bank) were cultured in Dulbecco’s modified Eagle’s medium (DMEM; Gibco, USA) supplemented with 10% fetal bovine serum (FBS; Gibco, USA) under standard conditions (37 °C, 5% CO_2_). The cells were subcultured at 80–90% confluence and assigned to two experimental groups: (1) the high-glucose (HG) group, which contained DMEM supplemented with 30 mmol/L glucose, and (2) the control group, which contained DMEM supplemented with physiological glucose (5 mmol/L). Both groups were maintained under identical culture conditions for 48 h before proceeding with downstream assays.

### ​Ultrastructural analysis of the samples​

#### ​Conventional scanning electron microscopy

Myocardial samples were fixed overnight at 4 °C in 2.5% glutaraldehyde, followed by four 10-minute washes in phosphate buffer (PB, pH 7.4). Sequential dehydration was performed using graded ethanol (50%, 70%, 80%, 90%, and 100%) and tert-butanol (10 min per step). The samples were then subjected to cryovacuum drying with tert-butanol and imaged using a field-emission scanning electron microscope (SEM, SU8010, Hitachi, Japan) at an accelerating voltage of 2 kV and a working distance of 8 mm to capture secondary electron signals.

#### ​Dimethyl sulfoxide (DMSO) cryofracture SEM​

Tissues were fixed in a dual fixative (2.5% glutaraldehyde + 2.5% paraformaldehyde) at 4 °C overnight, rinsed in 0.1 M PB, and processed *via* an osmium-DMSO-osmium cryofracture protocol using liquid nitrogen. The dehydration, drying, and imaging steps followed the same methodology outlined previously.

#### Serial sectioning and 3D reconstruction

(1) Sample preparation: Fixed myocardial tissues underwent postfixation in 1% osmium tetroxide/1.5% potassium ferrocyanide for 1 h at room temperature, followed by thiocarbohydrazide (1%, 30 min, room temperature) and secondary osmication with 2% OsO_4_ for 1 h at room temperature. Contrast enhancement was achieved with 1% uranyl acetate overnight at 4 °C. Dehydration in graded ethanol and acetone was followed by progressive resin infiltration with Spon 812 epoxy (acetone: resin ratios of 3:1, 1:1, and 1:3; 2–3 h per step) and final polymerization at 60 °C for 48 h. (2) Serial sectioning and imaging: Ultrathin Sect. (70 nm) were cut using an ultramicrotome (EM UC7, Leica, Germany), collected on silicon wafers, and imaged in backscattered electron mode *via* SEM (2 kV, 3 mm working distance). (3) 3D reconstruction: Serial images were aligned, segmented, and reconstructed using Amira 3D software (Thermo Fisher Scientific). Mitochondrial morphometrics, such as volume and anisotropy, were quantified using the *Label Analysis* module, with 3D surface rendering performed through the *Generate Surface* and *Surface View* utilities.

#### Statistical analysis of mitochondrial cristae and MAM-related parameters

Statistical analyses were performed using the 2D images used for 3D reconstruction. Mitochondria were scored based on cristae integrity: (1) intact lamellar cristae (integrity > 90%); (2) partial cristae loss (30–70% retained); (3) cristae disintegration (detectable < 10%). Only mitochondria with clearly discernible cristae were selected for statistical analysis. The “Threshold” function in ImageJ was used to generate grayscale images of mitochondrial cristae morphology. The parameters of mitochondrial cristae width, cristae spacing, and cristae junction width were recorded using ImageJ measurement tools [11]. MAM-related parameters were analyzed using Amira software. Mitochondria and ER were segmented, and the lengths of MAM contact sites, as well as the perimeter of both mitochondria and ER, were calculated using the *Crofton Perimeter* and *InsideLength* modules within the *Label Analysis* module.

### Western blot analysis​

Total protein was extracted from mouse myocardial tissue using a total protein extraction kit, supplemented with a Protease Inhibitor Cocktail. Protein concentration was quantified using a BCA protein quantification kit (Pierce, USA). SDS-PAGE gels were prepared with 8–12% separating gels and 5% stacking gels. For each sample, 60 µg of total protein (10–15 µL per well) was loaded into the wells. Electrophoresis was carried out at 60 V for the stacking gel and 80 V for the separating gel for approximately 2 h. PVDF membranes (IPVH00010, Millipore, USA) were pre-wetted in methanol for 20 s and then equilibrated in Tris-Glycine transfer buffer (containing 5% methanol) for at least 5 min. SDS-PAGE gels were equilibrated in Tris-Glycine transfer buffer for at least 30 min. Wet transfer was performed at a constant voltage of 100 V under cooling conditions for 2 h. After transfer, the membranes were placed in T-TBS (containing 5% BSA) for 1 h of room temperature blocking, followed by three 5-minute washes with T-TBS. Primary antibodies were diluted in T-TBS (3% BSA) and incubated with the membranes overnight at 4 °C. Membranes were then washed four times with T-TBS for 5 min each. The primary antibodies used were as follows: Mfn1 (1:1000, #ab221661; Abcam, UK), Mfn2 (1:1000, #9482; Cell Signaling Technology, USA), Opa1 (1:2000, #27733-1-AP; Proteintech, China), Drp1 (1:1000, #5391; Cell Signaling Technology), p-Drp1(Ser616) (1:1000, #ab314755; Abcam, UK), PINK1 (1:500, #PA1-16604; Thermo Fisher), Parkin (1:1000, #14060-1-AP; Proteintech), and VDAC1 (1:1000, #ab306581; Abcam, UK) as loading controls. Secondary antibodies were diluted in T-TBS (3% BSA) and incubated with the membranes for 1 h at room temperature, followed by five 5-minute washes with T-TBS. ECL detection was carried out using SuperSignal^®^ West Dura Extended Duration Substrate according to the manufacturer’s instructions. One milliliter of ECL working solution was added to the membrane, incubated for 1 min at room temperature, and excess reagent was removed. The membrane was then sealed with plastic wrap. X-ray films were exposed for 5–10 min in a dark box, followed by development and fixation. Band grayscale values were quantitatively analyzed using ImageJ software (NIH, USA).

### ​Mitochondrial function assessment​

#### ​Assay of total ATP content in myocardial tissue

Fresh myocardial tissues were homogenized *via* ultrasonic disruption, and the supernatants were collected after centrifugation (12,000 ×g, 10 min, 4 °C). The ATP content was measured using an ATP Assay Kit (Nanjing Jiancheng Bioengineering Institute, #A095-1-1), with absorbance read at 636 nm using a UV-2550 spectrophotometer (Shimadzu). The ATP content (µmol/g tissue) was calculated as follows:

$$\begin{aligned} & {\text{ATP}}~{\text{content}}\left( {\frac{{\mu {\text{mol}}}}{{\text{g}}}} \right){\text{ = }} \\ & \frac{{{\text{Ameasured - Acontrol}}}}{{{\text{Astandard - Ablank}}}} \\ & \times {\text{Cstand}} \times N \div \left( {\frac{{\text{W}}}{{{\text{Vtotal}}~{\text{sample}}}}} \right) \\ \end{aligned}$$,

where *A*measured, *A*control, *A*standard, and *A*blank​denote the absorbances of the test sample, blank control, ATP standard, and standard blank, respectively; *C*standard is the ATP standard concentration (µmol/L); *N* represents the dilution factor; *W* is the tissue weight (g); and *V*total sample is the total homogenate volume (L).

#### Mitochondrial membrane potential (ΔΨm) assay in myocardial tissue

Mouse heart tissue was dissected into small pieces and transferred to a culture dish containing 2 mL of pre-chilled PBS (4 °C). The tissue was minced using sterile surgical scissors, followed by the addition of 1 mL of 10% collagenase II (Sigma, C6885) and 2.5 mL of 10% collagenase IV (Sigma, C5138). The mixture was incubated in a 37 °C shaker at 15 rpm for 30 min to facilitate tissue digestion. After digestion, the sample was sequentially filtered through 70 μm and 30 μm cell strainers to collect the filtrate, forming a cell suspension. Red blood cells in the suspension were lysed using a red blood cell lysis solution. The cell count was determined, and 300,000 cells were collected, resuspended in culture medium, and gently vortexed with 0.5 mL of JC-1 staining working solution. The suspension was incubated at 37 °C in a cell culture incubator for 20 min. Following incubation, the cells were centrifuged at 600 g for 3–4 min at 4 °C, the supernatant was discarded, and the pellet was washed twice with 1X JC-1 staining buffer. The cells were then resuspended in an appropriate volume of 1X JC-1 staining buffer and analyzed for fluorescence using a flow cytometer. Data processing was performed with NovoExpress software, with gating positions determined by using negative and positive control tubes. Quantitative analysis was conducted by calculating the percentage of cells within the gated region for each sample. Three samples were tested, and the mean values were reported.

#### ​Assay of mitochondrial respiratory chain complex activities in myocardial tissue​

The activities of OXPHOS complexes (I–V) were assessed using CheKine™ assay kits (Abbkine, #KTB1850, KTB1860, KTB1870, KTB1880, KTB1890). Mitochondria were incubated with substrate-specific reagents, and absorbance changes were monitored at wavelengths ranging from 340 to 600 nm (depending on the specific complex) using a Varioskan LUX microplate reader (Thermo Fisher). Activity calculations were performed using the provided formulas for each complex. The assay involved multiple sequential steps to ensure precise enzyme activity measurement. Initially, mitochondria were isolated through homogenization and centrifugation. Specific substrates for the target enzymes were then added according to the manufacturer’s guidelines, and the mixture was incubated at 37 °C for a designated time. The colorimetric changes in the reaction mixture served as indicators of enzyme activity, which were subsequently used to calculate the activity of mitochondrial respiratory chain complexes [12, 13].

#### Detection of MtDNA copy number in myocardial tissue

Total DNA from myocardial tissue was extracted using a tissue/cell genomic DNA rapid extraction kit (Aidlab, Cat. No. DN07). Mitochondrial DNA (mtDNA) copy number was quantified *via* quantitative real-time PCR (qPCR). The total reaction volume for qPCR was 10 µL, containing 5 µL of SYBR Green qPCR Master Mix, 2 µL of ddH_2_O, 2 µL of primers (1 µL forward primer + 1 µL reverse primer), and 1 µL of DNA template. The primers for the mtDNA target gene *ND1* were as follows: forward primer 5′-TAGAACGCAAAATCTTAGGGT-3′, reverse primer 5′-ATAATTTTATGGCGTCTGCAA-3′. The primers for the reference gene *HBB* were as follows: forward primer HBB-F: 5′-CCTGGCTCACAAGTACCAC-3′, reverse primer HBB-R: 5′-TCATTTTGCCAACAACTGACAGA-3′. DNA amplification was performed using a two-step method with the following conditions: (1) pre-denaturation at 95 °C for 10 min; (2) 95 °C for 10 s, 60 °C for 2 min, 40 cycles. Relative gene expression was calculated using the 2^–ΔΔCt^ method, with each sample tested in triplicate.

#### Mitochondrial Ca²⁺ detection by flow cytometry

Mouse myocardial tissue was processed to prepare a single-cell suspension as described previously. The suspension was resuspended in PBS to a final concentration of 1 × 10^6^ cells/mL. To this, 50 µL of Rhod-2 staining solution was added, and the mixture was incubated at 37 °C for 30 min. After incubation, cells were centrifuged at 600 × g for 4 min at 4 °C to pellet. The pellet was washed twice with PBS: 1 mL of PBS was added to resuspend the cells, followed by centrifugation at 600 × g for 4 min at 4 °C, and the supernatant was discarded. This washing procedure was repeated once more. The cells were then resuspended in 1 mL of PBS, and fluorescence signals indicating calcium ion levels were detected using a flow cytometer with an excitation wavelength of 552 nm and an emission wavelength of 581 nm. A Ca^2+^ Up (CaUp)-treated sample was used as a positive control, and thresholds were set to exclude cell debris. Flow cytometry data were analyzed using NovoExpress software, with each sample measured in triplicate.

#### Determination of superoxide dismutase (SOD) activity in myocardial tissue

Myocardial tissue sample (0.1 g) was homogenized in ice-cold normal saline (1:9, w/v) using a homogenizer, followed by centrifugation at 4000 × g for 10 min. The supernatant was collected for the SOD assay. SOD activity in the myocardial tissue was measured using an SOD Assay Kit (WST-1 method) (Nanjing Jiancheng Bioengineering Institute, Cat. No. A001-3). The reagent addition scheme was as follows: (1) Control well: 20 µL of distilled water + 20 µL of enzyme working solution + 20 µL of enzyme dilution + 200 µL of substrate application solution; (2) Test well: 20 µL of sample + 20 µL of enzyme working solution + 200 µL of substrate application solution; (3) Control blank well: 20 µL of distilled water + 20 µL of enzyme dilution + 200 µL of substrate application solution; (4) Test blank well: 20 µL of sample + 20 µL of enzyme dilution + 200 µL of substrate application solution. After mixing, the samples were incubated at 37 °C for 20 min, and absorbance was measured at 450 nm using a microplate reader (Thermo Fisher, Varioskan LUX). The following formulae were used: SOD activity

$$\begin{aligned} & \left( {{\text{U}}/{\text{mg prot}}} \right){\text{ }} = \\ & \frac{{{\text{SODinhibition}}~{\text{rate}}}}{{{\text{50}}\% }} \times \frac{{{\text{0}}{\text{.24mL}}}}{{{\text{0}}{\text{.02mL}}}} \\ & \div {\text{protein}}~{\text{concentration}}~{\text{of}}~{\text{sample}}~({\text{mg/mL}}); \\ \end{aligned}$$

$$\begin{aligned}& {\text{SOD inhibition rate }}\left( \% \right) = \\ & \frac{{\left( {{\text{Acontrol}} - {\text{Acontrol}}~{\text{blank}}} \right) - \left( {{\text{Atest}} - {\text{Atest}}~{\text{blank}}} \right)}}{{\left( {{\text{Acontrol}} - {\text{Acontrol}}~{\text{blank}}} \right)}} \\ \end{aligned}.$$

#### Live-cell oxygen consumption rate (OCR) analysis​

The real-time OCR of H9c2 cardiomyocytes was measured using an O2k high-resolution respirometer (Oroboros Instruments). The OCR assay buffer was composed of DMEM low-glucose medium (GIBCO) supplemented with 10% FBS (Sijiqing). For each group, 500,000 cells were normalized and used for the experiment. A titration protocol involving Oligomycin, FCCP, Rot, and Antimycin A was applied, with specific working solutions prepared as follows: Oligomycin at 0.01 mM (solvent: anhydrous ethanol, GLPBIO, GC16533); FCCP at 1 mM (solvent: DMSO, GLPBIO, GC14328); Antimycin A at 5 mM (solvent: DMSO, GLPBIO, GC49360); and Rot at 1 mM (solvent: DMSO, GLPBIO, GC16775). The following parameters of viable cells were measured: basal respiration, ATP-linked respiration, proton leakage, maximal respiratory capacity, spare respiratory capacity, and non-mitochondrial respiration.

### Statistical analysis and data visualization

Statistical analyses were performed using GraphPad Prism software (La Jolla, CA, USA). The Shapiro-Wilk test was used to assess data normality. If the data followed a normal distribution, the t-test was applied; otherwise, the Mann-Whitney test was used. A significance level of *p* < 0.05 was considered significant (*), with higher levels of significance (*p* < 0.01, *p* < 0.001, and *p* < 0.0001) represented as (**, ***, and ****), respectively.

## Results​

### ​Evaluation of blood glucose, histopathology, and cardiac function in DCM mice

**Fig. 1:**
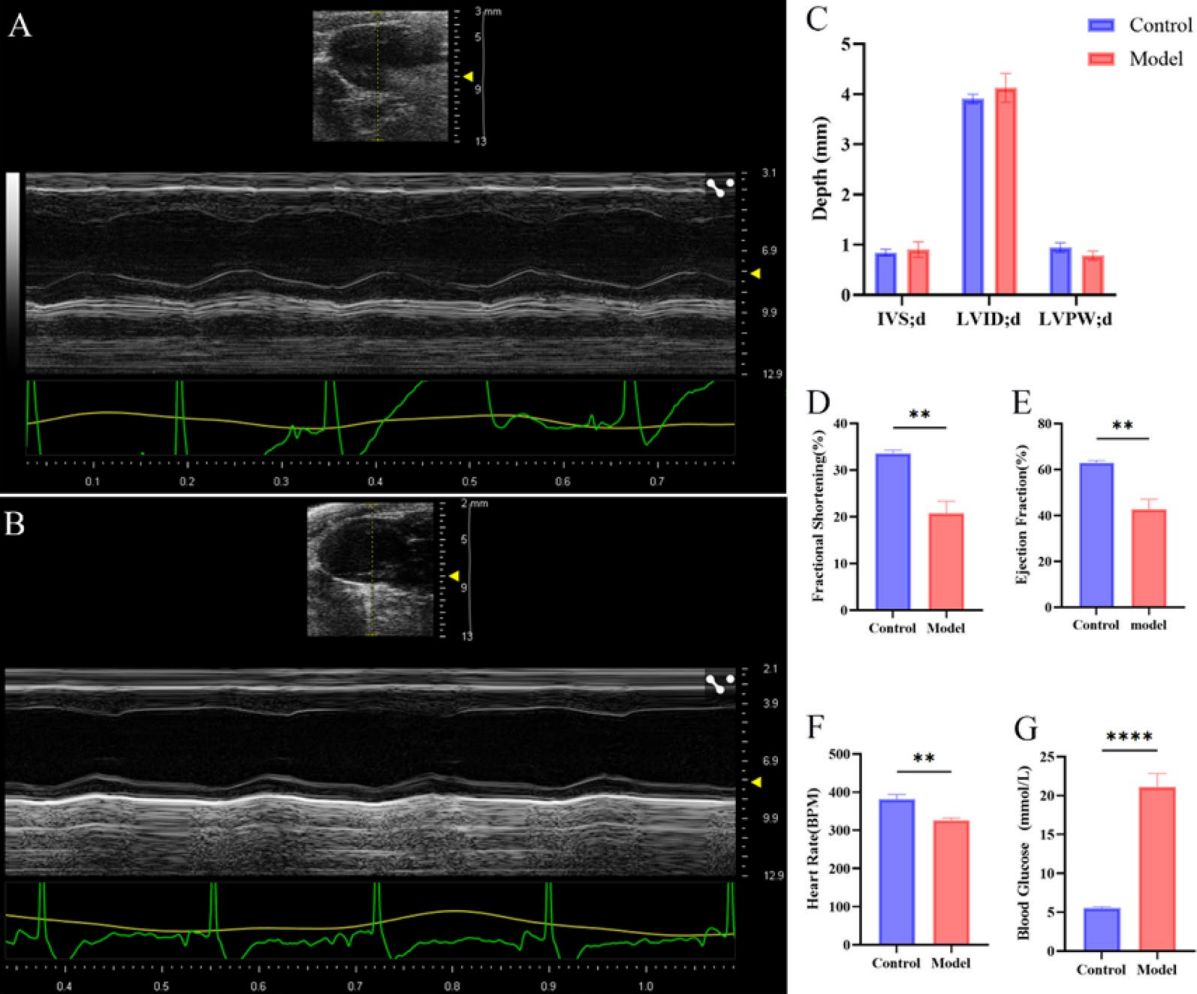
Echocardiographic evaluation of cardiac function in mice Note: **(A)** Representative echocardiogram of a mouse heart from the control group. *n* = 3 per group. **(B)** Representative echocardiogram of a mouse heart from the model group. *n* = 3 per group. **(C)** Measurements of the diastolic interventricular septum (IVSd), left ventricular internal diameter (LVIDd), and left ventricular posterior wall thickness (LVPWd) in the control and model groups. *n* = 3 per group. **(D)** Fractional shortening (FS) in the control and model groups. *n* = 3 per group. **(E)** Ejection fraction (EF) in the control and model groups. *n* = 3 per group. **(F)** Heart rate in the control and model groups. *n* = 3 per group. **(G)** Blood glucose levels in the control and model groups. *n* = 10 per group. Data are presented as mean ± standard deviation. Significance levels: **p < 0.01, ****p < 0.0001.

**Fig. 2:**
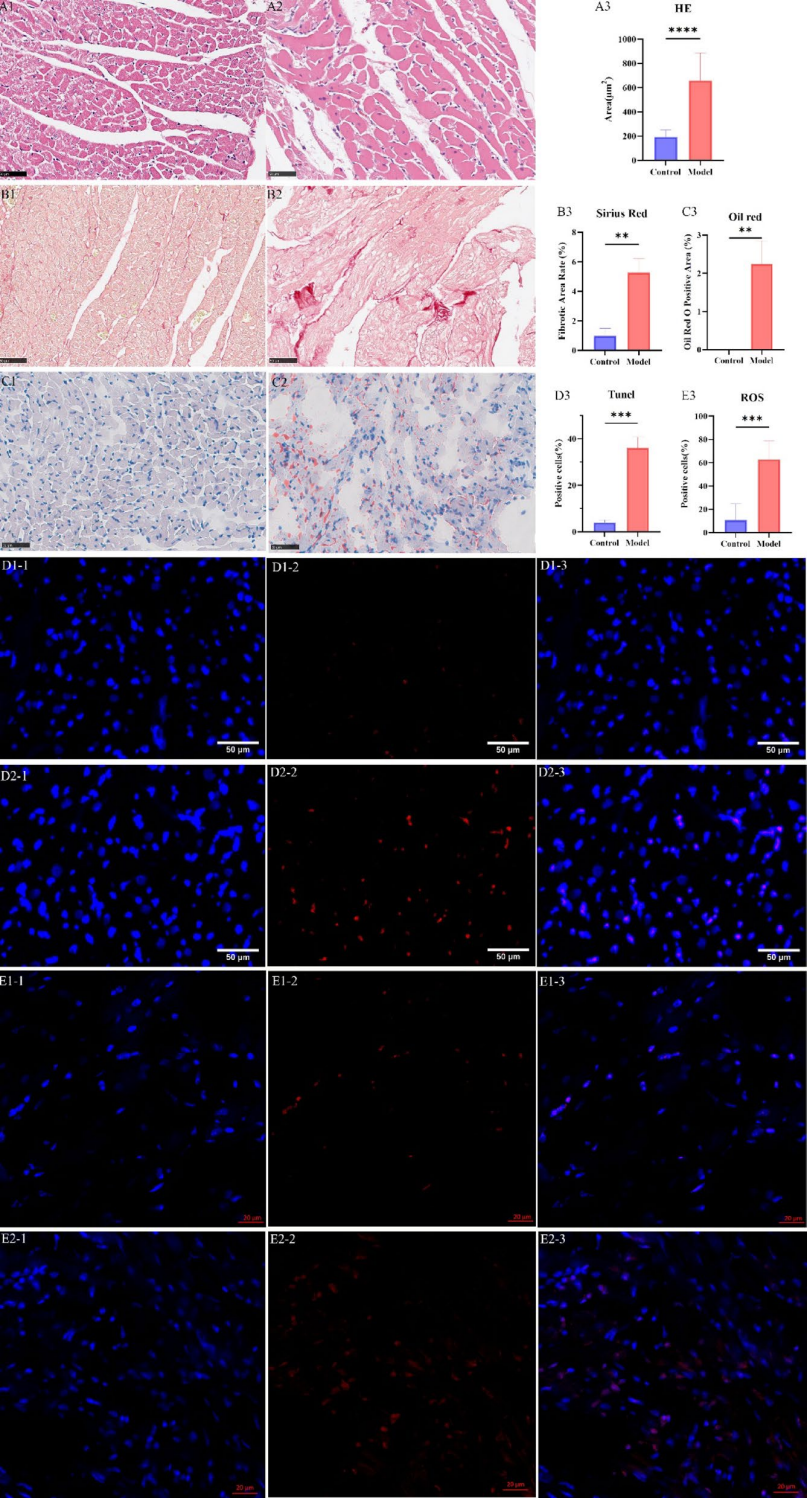
Histopathological images of myocardial tissue in mice Note: **(A)** H&E staining of myocardial tissue. *n* = 3 per group. A1. Control group. A2. DCM group. A3. Statistical analysis of cardiomyocyte area between the two groups. **(B)** Sirius Red staining of myocardial tissue. *n* = 3 per group. B1. Control group. B2. DCM group. B3. Statistical analysis of the proportion of fibrotic area relative to total area between the two groups. **(C)** Oil Red O staining of myocardial tissue. *n* = 3 per group. C1. Control group. C2. DCM group. C3. Statistical analysis of the area of oil red O-positive regions relative to the total area between the two groups. **(D)** TUNEL staining results. *n* = 3 per group. D1. Control group. D2. DCM group. D1-1 and D2-1: DAPI-stained cell nuclei (blue). D1-2 and D2-2: TUNEL-positive apoptotic cell nuclei (green). D1-3 and D2-3: Merged images. D3. Statistical analysis of the proportion of apoptotic cells relative to total cells between the two groups. **(E)** ROS staining results. *n* = 3 per group. E1. Control group. E2. DCM group. E1-1 and E2-1: DAPI-stained cell nuclei (blue). E1-2 and E2-2: ROS-positive cells (red). E1-3 and E2-3: Merged images. E3. Statistical analysis of the proportion of ROS-positive cells relative to total cells between the two groups. Scale bar = 50 μm. Data are presented as mean ± standard deviation. Significance levels: **p < 0.01, ***p < 0.001, ****p < 0.0001.

Blood glucose measurements revealed significantly elevated FBG levels in the DCM mice compared to controls (Fig. 1G; *p* < 0.0001). Quantitative echocardiographic assessment confirmed significant reductions in LVEF, LVFS, and heart rate in the DCM group compared to controls (Figs. 1D-F; *p* < 0.01; Supplemental Video S1, S2) Histological analyses using H&E, Sirius Red, and Oil Red O staining further revealed distinct pathological features in the DCM group: cardiomyocytes exhibited hypertrophy and degeneration, myocardial disarray, increased fibrosis, and lipid accumulation, all of which were significantly more pronounced compared to the control group (Fig. 2A-C). TUNEL staining results showed a significantly higher apoptosis rate in the DCM group (Fig. 2D), alongside a marked increase in the percentage of ROS-positive cells (Fig. 2E). These results collectively indicated a substantial impairment of cardiac function in the diabetes-induced mouse model.

### Ultrastructural visualization of partial structures in myocardial tissue of the control group

**Fig. 3:**
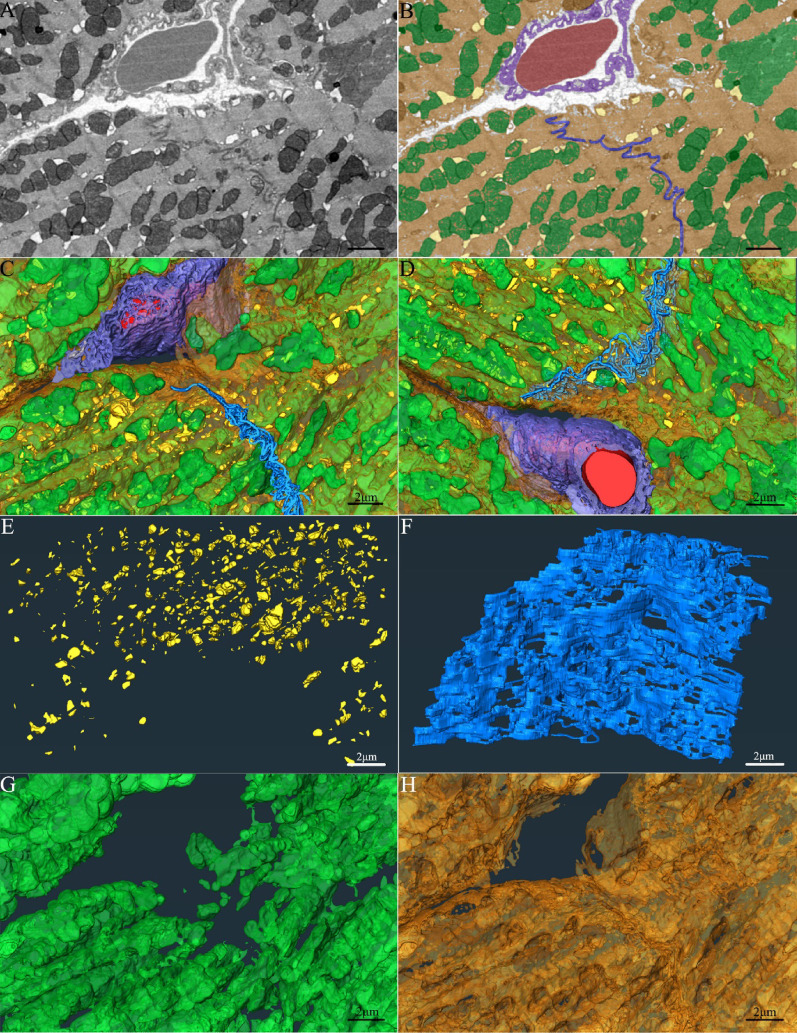
Ultrastructural and three-dimensional analysis of the myocardial architecture Note: **(A)** 2D image of myocardial tissue. **(B)** Segmented subcellular structures. **C-D.** 3D renderings from different perspectives. **E.** 3D reconstruction of T-tubules. **F.** 3D reconstruction of intercalated discs. **G.** 3D reconstruction of mitochondria. **H.** 3D reconstruction of myofilaments. Color key: Mitochondria (green), T-tubules (yellow), intercalated discs (blue), red blood cells (red), blood vessel walls (purple), myofilaments (orange). Scale bar = 2 μm.

2D images (Fig. 3A) clearly illustrate key ultrastructural components of cardiomyocytes, including parallel-aligned myofibrils interspersed with mitochondria, T-tubule systems, intercalated discs, and capillaries. The segmentation of major subcellular structures (Fig. 3B) and 3D reconstruction (Fig. 3C-G; Supplemental Video S3) revealed that the mitochondria were densely packed and regularly arranged within cardiomyocytes, with T-tubules closely associated with mitochondrial clusters. Intercalated discs exhibited a characteristic stepped and highly folded morphology.

### Statistical analysis of mitochondrial parameters across different mitochondrial subpopulations in the myocardial tissue of control mice

**Fig. 4:**
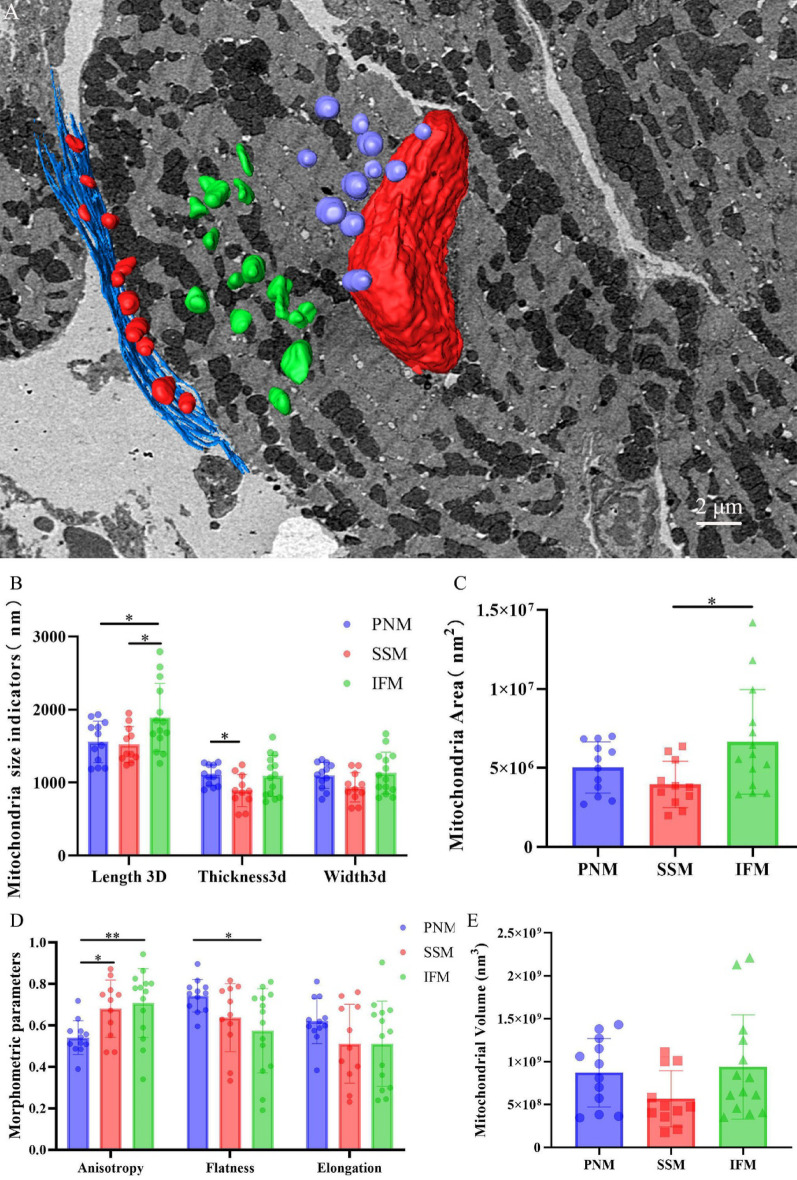
3D ultrastructural images and three-dimensional morphological indicators of mitochondrial subpopulations Note: **(A)** Three-dimensional display of mitochondrial subpopulations. Color key: SSM (red), IFM (green), PNM (puple), Scale bar = 2 μm. **(B)** Statistical analysis of mitochondrial size indicators (length, width, and thickness) in different subpopulations (*n* = 3 per group). **(C)** Statistical analysis of mitochondrial surface area in different subpopulations. *n* = 3 per group. **(D)** Statistical analysis of mitochondrial morphometric parameters (anisotropy, flatness, elongation) in different subpopulations. *n* = 3 per group. **(E)** Statistical analysis of mitochondrial volume in different subpopulations. *n* = 3 per group. Data presented as mean ± standard deviation. Significance levels: *p < 0.05, **p < 0.01.

3D morphometric analysis of mitochondrial subpopulations in cardiomyocytes revealed significant spatial heterogeneity (Fig. 4A; Supplemental Video S4). Mitochondria were categorized into three subpopulations based on their spatial localization: subsarcolemmal mitochondria (SSM), interfibrillar mitochondria (IFM), and perinuclear mitochondria (PNM). 3D visualization showed that the IFM exhibited marked morphological diversity, including spherical, elongated, and irregular shapes, whereas the SSM and PNM were predominantly spherical.

Metric comparisons revealed that the length of IFM (1.89 × 10^3^ ± 0.47 × 10^3^ nm) was significantly greater than that of both SSM and PNM (*p* < 0.05). No significant differences in width or thickness were observed among the three subpopulations, with all measurements approximating 1 × 10^3^ nm (Fig. 4B). The mean surface area of IFM (6.65 × 10^6^ ± 3.32 × 10^6^ nm^2^) was significantly larger than SSM (3.96 × 10^6^ ± 1.46 × 10^6^ nm^2^; *p* < 0.05; Fig. 4C), although no differences in volume were observed (Fig. 4E). Further analysis of three morphometric parameters (anisotropy, flatness, and elongation) revealed that IFM displayed significantly greater anisotropy than both PNM (*p* < 0.01) and SSM (*p* < 0.05). In contrast, PNM exhibited greater flatness compared to IFM (*p* < 0.05), while no significant differences in elongation values were observed across the groups (Fig. 4D).

### Effects of HG on the mitochondrial ultrastructure of H9c2 cardiomyocytes​

**Fig. 5:**
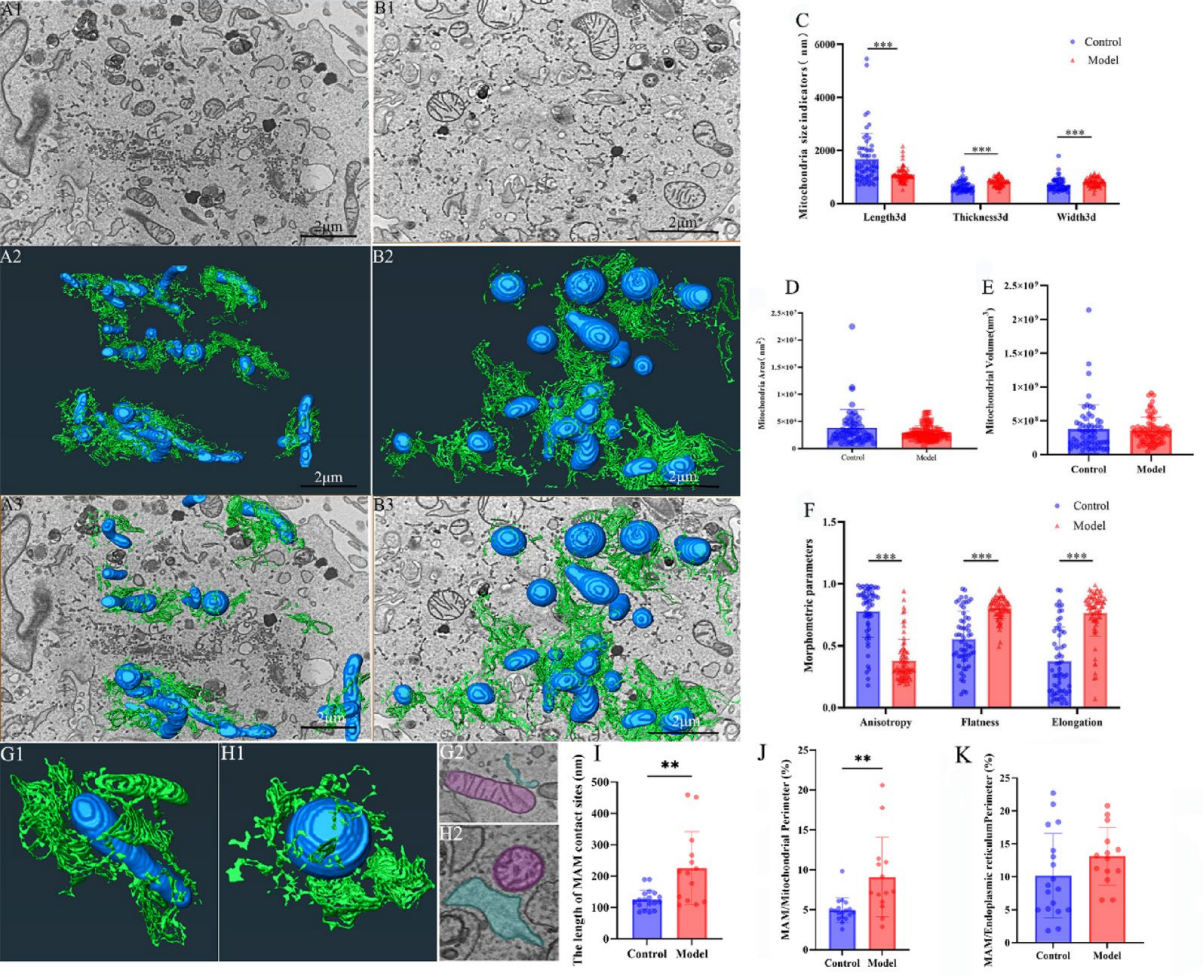
Effects of HG on mitochondrial morphology and mitochondria-associated membranes (MAM) in H9c2 cardiomyocytes Note: **(A)** Control group. **(B)** DCM group. A1, B1: Two-dimensional images of H9c2 cells. A2, B2: 3D rendering of mitochondria and the ER. Mitochondria (blue), ER (green). A3, B3: Overlay images of 2D and 3D structures. **C.** Mitochondrial size indicators (length/width/thickness, *n* = 3 per group). **D.** Mitochondrial surface area measurements. *n* = 3 per group. **E.** Morphometric parameters (anisotropy/flatness/elongation, *n* = 3 per group). **F.** Mitochondrial volume analysis. *n* = 3 per group. **G.** Control group. **H.** DCM group. GI, H1: 3D visualization of mitochondria-associated membranes (MAM). G2, H2: 2D visualization of mitochondria-associated membranes (MAM). **I.** Length of MAM contact sites. *n* = 3 per group. J. MAM/mitochondrial perimeter. *n* = 3 per group. K. MAM/ER perimeter. *n* = 3 per group. Data are presented as mean ± standard deviation. Significance levels: **p < 0.01, ***p < 0.001

3D morphometric analysis revealed that, compared to controls, mitochondria in the HG-treated H9c2 cardiomyocytes exhibited a reduced prevalence of elongated morphologies, with a predominant shift toward shortened, spherical forms under hyperglycemic conditions. No significant differences in ER morphology were observed between the two groups (Fig. 5A3, B3; Supplemental Video S5, S6). 3D morphological indicators showed that the mitochondrial Length3D in the control group was 1.68 × 10^3^ ± 0.97 × 10^3^ nm, which was significantly greater than that in the model group (1.10 × 10^3^ ± 0.27 × 10^3^ nm; *p* < 0.0001). In contrast, Thickness3D and Width3D exhibited opposite trends. The Thickness3D in the control group was 6.53 × 10^2^ ± 2.01 × 10^2^ nm, which increased to 8.27 × 10^2^ ± 1.54 × 10^2^ nm in the model group (*p* < 0.0001). The Width3D in the control group was 7.21 × 10^2^ ± 2.39 × 10^2^ nm, which increased to 8.25 × 10^2^ ± 1.54 × 10^2^ nm in the model group (*p* < 0.01) (Fig. 5C). The mitochondrial Area3D in the control group (3.81 × 10^6^ ± 3.36 × 10^6^ nm^2^) was significantly larger than that in the model group (2.88 × 10^6^ ± 1.31 × 10^6^ nm^2^; *p* < 0.05) (Fig. 5D). Mitochondrial Volume3D did not show significant differences between the control and model groups (Fig. 5E). Analysis of morphometric parameters revealed that the anisotropy value of the control group (0.78 ± 0.21) was significantly greater than that of the model group (0.38 ± 0.17; *p* < 0.001). The flatness value of the control group (0.55 ± 0.22) was significantly lower than that of the DCM group (0.81 ± 0.09; *p* < 0.001). The elongation value of the control group (0.38 ± 0.27) was significantly lower than that of the model group (0.76 ± 0.19; *p* < 0.001) (Fig. 5F). Additionally, a statistical analysis of mitochondria-associated membrane (MAM) parameters was conducted. The results showed that the length of MAM contact sites in the model group (2.25 × 10^2^ ± 1.17 × 10^2^ nm) was significantly greater than that in the control group (1.25 × 10^2^ ± 0.31 × 10^2^ nm) (*p* < 0.01). The MAM/mitochondrial perimeter ratio in the model group was 9.09% ± 4.99%, significantly higher than in the control group (4.95% ± 1.53%) (*p* < 0.01). For the MAM/ER perimeter, the model group displayed 13.11% ± 4.38%, while the control group exhibited 10.16% ± 6.42%, with no significant difference observed between the two groups (Fig. 5G-K).

### Ultrastructural investigation of myocardial mitochondria in DCM mice​

This study conducted a comprehensive analysis of mitochondrial ultrastructure in DCM model mice using three SEM techniques: (1) conventional SEM sample preparation and imaging; (2) DMSO-assisted freeze-fracture SEM, which rapidly freezes and mechanically fractures tissues to expose mitochondrial cristae structures, facilitating high-depth 3D surface imaging; and (3) a combination of serial ultrathin sectioning with 3D reconstruction, enabling detailed 3D visualization and quantitative analysis of morphometric parameters.

### Freeze-fracture SEM observations of mitochondria

**Fig. 6:**
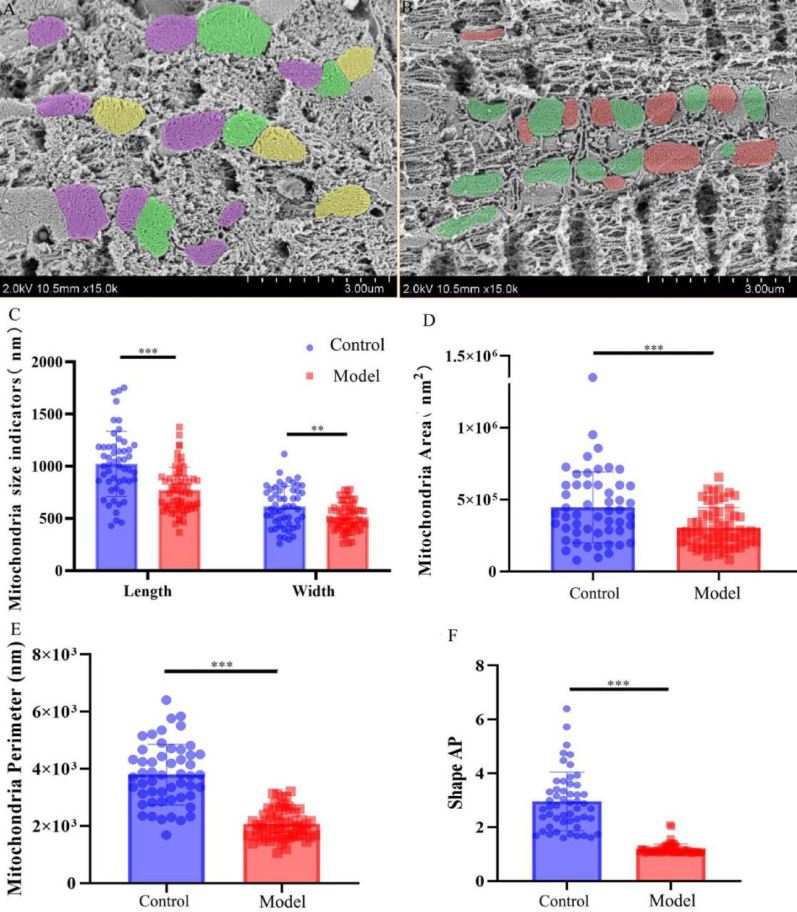
Three-dimensional surface imaging of myocardial mitochondria and two-dimensional morphological indicators Note: **(A)** Three-dimensional surface imaging of myocardial tissue in the control group. **(B)** Three-dimensional surface imaging of myocardial tissue in the DCM group. **(C)** Statistical analysis of mitochondrial size indicators (length/width, *n* = 3 per group). **(D)** Mitochondrial area measurements. *n* = 3 per group. **(E)** Mitochondrial perimeter measurements. *n* = 3 per group. **(F)** Mitochondrial shape AP analysis. *n* = 3 per group. Data are presented as mean ± standard deviation. Significance levels: **p < 0.01, ***p < 0.001.

This method captures the fractured surface of samples, providing 3D surface scans, although the images remain two-dimensional for parameter quantification. SEM images of myocardial tissue from the control and DCM groups distinctly revealed myofilament, mitochondrial, and T-tubule structures (Fig. 6A, B). Quantitative analysis of color-annotated mitochondria showed that the mitochondrial length in the control group (1.02 × 10^3^ ± 0.31 × 10^3^ nm) was significantly greater than in the DCM group (0.77 × 10^3^ ± 0.22 × 10^3^ nm; *p* < 0.001). Similarly, mitochondrial width in the control group (0.61 × 10^3^ ± 0.19 × 10^3^ nm) was significantly greater than in the DCM group (0.52 × 10^3^ ± 0.13 × 10^3^ nm; *p* < 0.01) (Fig. 6C). The mitochondrial perimeter in the control group (3.80 × 10^3^ ± 1.06 × 10^3^ nm) was significantly greater than in the DCM group (2.07 × 10^3^ ± 0.52 × 10^3^ nm; *p* < 0.001) (Fig. 6E). The mitochondrial area in the control group (4.46 × 10^5^ ± 2.46 × 10^5^ nm^2^) was significantly greater than in the DCM group (3.06 × 10^5^ ± 1.38 × 10^5^ nm^2^; *p* < 0.001) (Fig. 6D). The mitochondrial ShapeAP in the control group (2.96 ± 1.09) was significantly greater than in the DCM group (1.18 ± 0.20; *p* < 0.001) (Fig. 6F).

### Three-dimensional reconstruction of mitochondria from serial sections and SEM observations

**Fig. 7:**
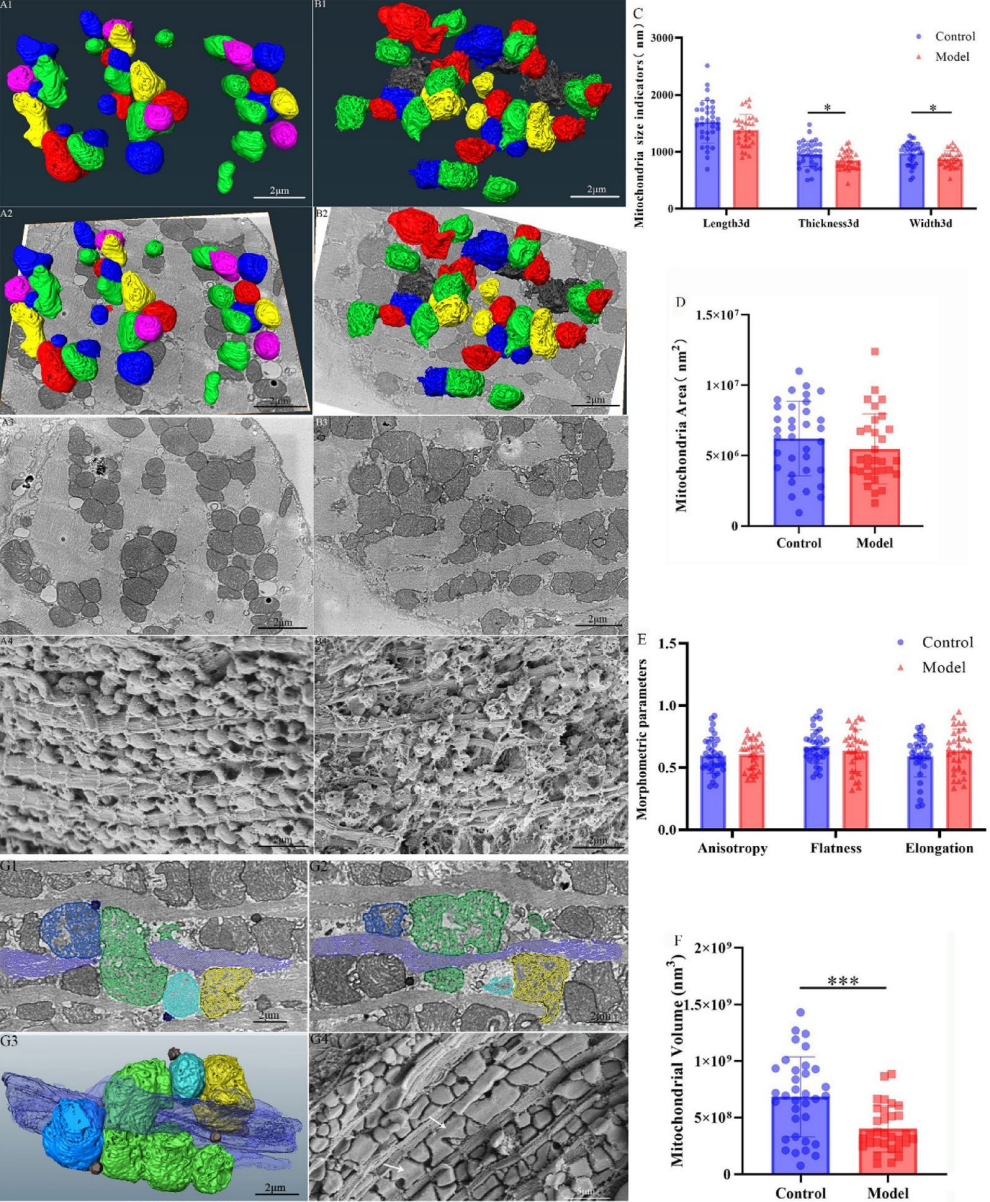
3D ultrastructural morphometry of myocardial mitochondria Note: **(A)** Control group. A1: 3D mitochondrial reconstruction; A2: 2D-SEM/3D overlay; A3: 2D images of myocardial tissue; A4: 3D surface images of myocardial mitochondria. **(B)** DCM model group. B1-B4: Corresponding analyses as in A. **(C)** Mitochondrial size indicators (length/width/thickness, *n* = 3 per group). **(D)** Mitochondrial surface area, *n* = 3 per group. **(E)** Morphometric parameters (anisotropy/flatness/elongation, *n* = 3 per group). **(F)** Mitochondrial volume, *n* = 3 per group. **(G)** Megamitochondria characterization. G1-G2: Two-dimensional (2D) visualization of megamitochondria in serial sections from different levels. G3: 3D rendering of megamitochondria. Scale bar = 2 μm. Color key: Myofilaments (purple), mitochondria (blue/green/cyan/gold), lipid droplets (black). G4: Three-dimensional surface imaging of myocardial mitochondria *via* SEM, white arrows indicate megamitochondria. Scale bar = 5 μm. Data are presented as mean ± standard deviation. Significance levels: *p < 0.05, ***p < 0.001.

Both conventional SEM and 3D reconstruction of serial sections revealed that mitochondria in the control group exhibited relatively smooth and rounded surfaces, whereas mitochondria in the DCM group presented rougher surfaces with occasional pore-like structures (Fig. 7A1, B1, A4, B4; Supplemental Video S7, S8). Further measurements of 3D morphological indicators showed that the 3D length of mitochondria in the control group (1.53 × 10^3^ ± 0.36 × 10^3^ nm) and in the DCM group (1.38 × 10^3^ ± 0.28 × 10^3^ nm) did not differ significantly. However, the Thickness3D of the control group (0.96 × 10^3^ ± 0.22 × 10^3^ nm) was significantly greater than that of the DCM group (0.85 × 10^3^ ± 0.16 × 10^3^ nm; *p* < 0.05). Similarly, the Width3D of the control group (0.97 × 10^3^ ± 0.19 × 10^3^ nm) was significantly greater than that of the DCM group (0.88 × 10^3^ ± 0.14 × 10^3^ nm; *p* < 0.05) (Fig. 7C). The 3D area values in the control group (6.21 × 10^6^ ± 2.64 × 10^6^ nm^2^) and DCM group (5.48 × 10^6^ ± 2.49 × 10^6^ nm^2^) were not significantly different (Fig. 7D). The 3D volume in the control group (6.84 × 10^8^ ± 3.54 × 10^8^ nm^3^) was significantly greater than in the DCM group (4.02 × 10^8^ ± 2.05 × 10^8^ nm^3^; *p* < 0.001) (Fig. 7F).

The morphometric parameters for anisotropy, flatness, and elongation were as follows: anisotropy in the control group was 0.60 ± 0.15, compared to 0.61 ± 0.12 in the DCM group; flatness was 0.67 ± 0.14 in the control group, and 0.64 ± 0.17 in the DCM group; elongation was 0.59 ± 0.17 in the control group and 0.64 ± 0.17 in the DCM group. None of these three parameters showed significant differences between the two groups (Fig. 7E). Despite the predominant ultrastructural features of mitochondrial fragmentation and volume reduction in the DCM group, 3D reconstruction unexpectedly revealed the presence of megamitochondria (Fig. 7G3; Supplemental Video S9). These abnormal mitochondrial structures, which were undetectable in conventional 2D TEM cross-sections, displayed the following 3D characteristics (Fig. 7G1, G2): a green-labeled mitochondrion with a maximum length of approximately 6 μm appeared to result from the fusion of four mitochondria. To further validate these findings, freeze-fracture SEM observations confirmed the presence of megamitochondria (Fig. 7G4).

### 3D reconstruction of MAM from serial sections

**Fig. 8:**
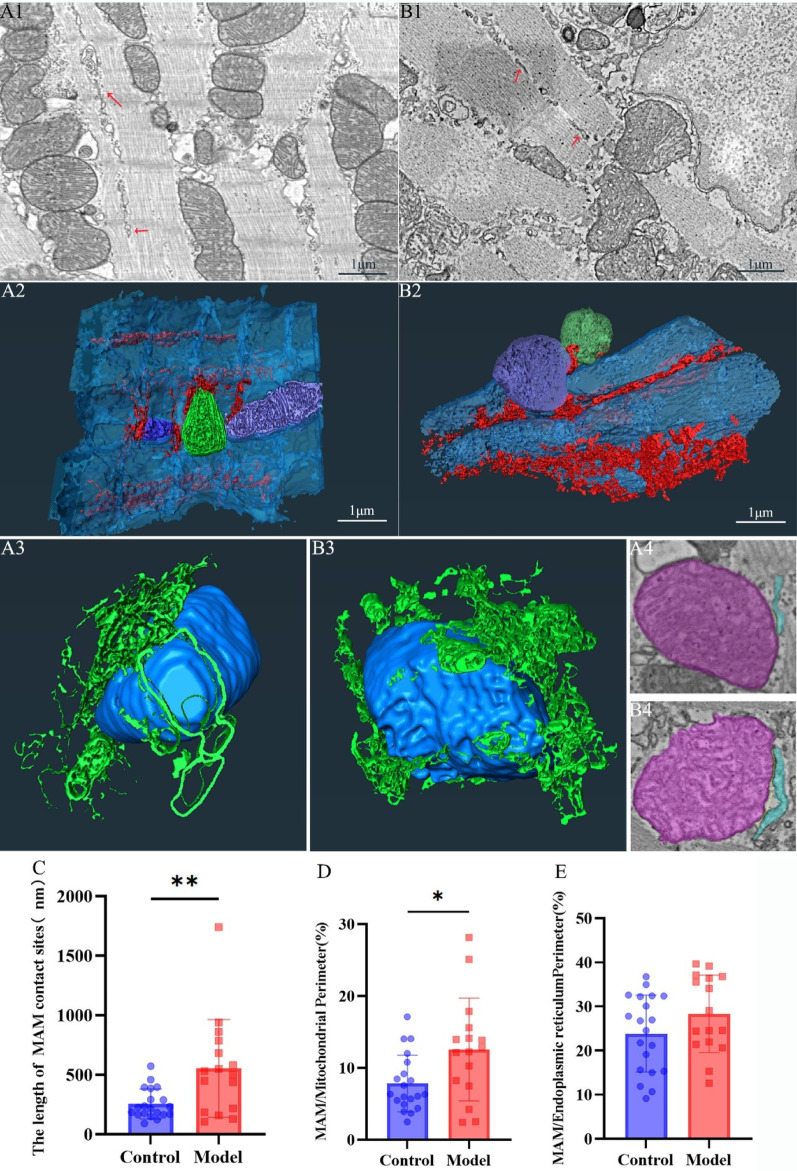
3D ultrastructural organization of the MAM Note: (**A**) Control group. A1: Representative 2D SEM micrograph of myocardial tissue, red arrows indicate SR; A2: 3D reconstruction of mitochondria (green/purple), sarcoplasmic reticulum (SR; red), and myofilaments (blue); A3: 3D rendering of MAM interfaces, mitochondria (blue), sarcoplasmic reticulum (green). A4: 2D display of MAM contacts, mitochondria (violet), sarcoplasmic reticulum (light green). (**B**) DCM group. B1-B4: Corresponding analyses as in A. (**C**) Length of MAM contact sites (µm). *n* = 3 per group. (**D**) MAM-mitochondrial contact perimeter ratio (%). *n* = 3 per group. (**E**) MAM-SR contact perimeter ratio (%). *n* = 3 per group. Data are presented as mean ± standard deviation. Significance levels: *p < 0.05, **p < 0.01.

SR, a specialized form of ER in cardiomyocytes, exhibits hollow tubular or filamentous structures in 2D cross-sections (Fig. 8A1, B1). 3D visualization reveals that the SR surrounds mitochondria and myofilaments, forming a complex network system. However, no significant structural differences were observed between the control and DCM groups (Fig. 8A2, B2; Supplemental Video S10, S11). Statistical analysis of MAM parameters from 2D sections showed that the length of MAM contact sites in the control group was 2.55 × 10^2^ ± 1.26 × 10^2^ nm, significantly lower than in the DCM group (5.54 × 10^2^ ± 4.11 × 10^2^ nm) (*p* < 0.01) (Fig. 8C). The MAM/mitochondrial perimeter ratio in the DCM group was 12.57% ± 7.15%, significantly higher than in the control group (7.83% ± 3.94%) (*p* < 0.05). For the MAM/ER perimeter, the DCM group displayed 28.35% ± 8.78%%, while the control group exhibited 23.87% ± 8.71%, with no significant difference observed between the two groups (Fig. 8E).

### Ultrastructural investigation of mitochondrial cristae​ in myocardial tissue

**Fig. 9:**
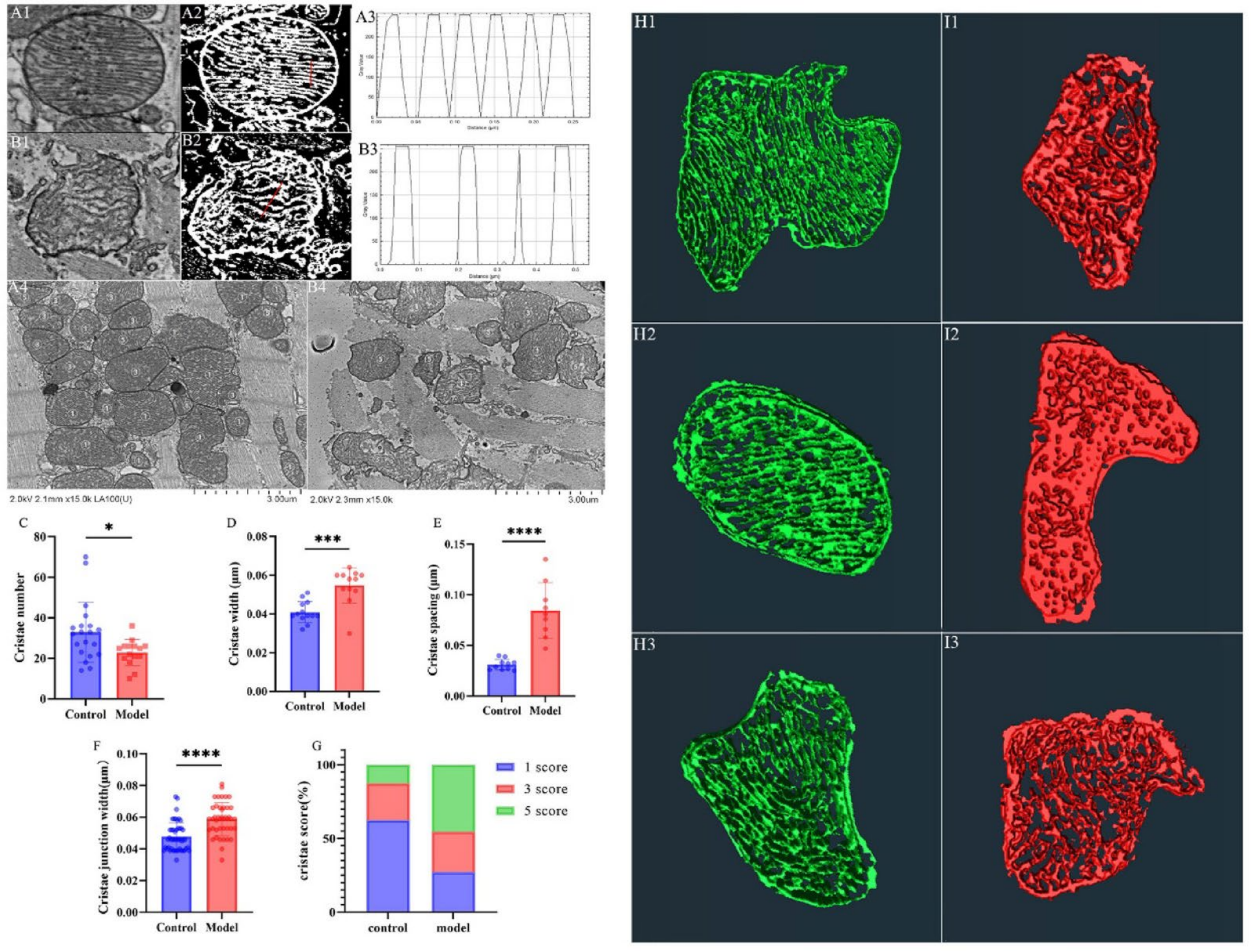
Quantitative analysis of mitochondrial cristae morphology in myocardial tissue Note: **(A)** Representative SEM images of control group myocardium. A1: Mitochondrial ultrastructure; A2: ImageJ-thresholded mitochondrial binary mask; A3: Cristae density analysis (red lines indicate line profiles); A4: Cristae integrity scoring scale (Grade 1–5). **(B)** SEM images of DCM group; B1-B4: Corresponding analyses as in A. **(C)** Cristae number per mitochondrion. **(D)** Cristae width. **(E)** Cristae spacing. **(F)** Cristae junction width. **(G)** Distribution of crista integrity scores. (1): Intact lamellar cristae (> 90% integrity). (3): Regional crista loss (30–70% preserved). (5): Cristae disintegration (< 10% detectable). (H) 3D cristae reconstruction (H1-H3: Control group); (I) 3D cristae reconstruction (I1-I3: DCM group). Data are presented as mean ± standard deviation. Significance levels: *p < 0.05, ***p < 0.001, ****p < 0.0001.

This study systematically examined pathological alterations in mitochondrial cristae structure in DCM through integrated 2D and 3D imaging analyses. 2D cross-sectional imaging revealed that the majority of cristae in the control group exhibited well-defined lamellar structures, while DCM mitochondria displayed a higher proportion of cristae with dissolution, disorganized arrangements, and even intramitochondrial vacuolization (Fig. 9A1, B1). Mitochondrial cristae width, spacing, junction width, and the number of cristae were quantified using ImageJ-based grayscale thresholding. Statistical analysis showed that the number of cristae in the control group (33 ± 14) was significantly higher than in the DCM group (23 ± 6) (*p* < 0.05). The width of the cristae in the control group (0.41 ± 0.005 μm) was significantly smaller than in the DCM group (0.55 ± 0.009 μm; *p* < 0.001). The crista spacing in the control group (0.031 ± 0.005 μm) was significantly smaller than that in the DCM group (0.084 ± 0.028 μm; *p* < 0.0001). Additionally, the crista junction (CJ) width in the control group (0.048 ± 0.009 μm) was significantly smaller than that in the DCM group (0.059 ± 0.010 μm) (*p* < 0.0001) (Fig. 9C-F). A crista integrity scoring system was established: Level 1 indicated intact lamellar cristae (> 90% structural integrity), Level 3 indicated regional crista loss (30–70% preserved), and Level 5 indicated crista disintegration (< 10% detectable structures) (Fig. 9A4, B4). Quantitative analysis revealed that Level 1 cristae were predominant in the control group (approximately 60%), while Level 5 cristae were most frequent in the DCM group (approximately 50%) (Fig. 9G). 3D visualization further confirmed these findings. In the control group, the cristae displayed orderly lamellar arrangements with stable spacing. In contrast, DCM cristae were largely unrecognizable, exhibiting chaotic arrangements and intracrystalline vacuoles, consistent with the 2D observations (Fig. 9H, I).

### Expression of proteins related to mitophagy and mitochondrial dynamics in myocardial tissue

**Fig. 10:**
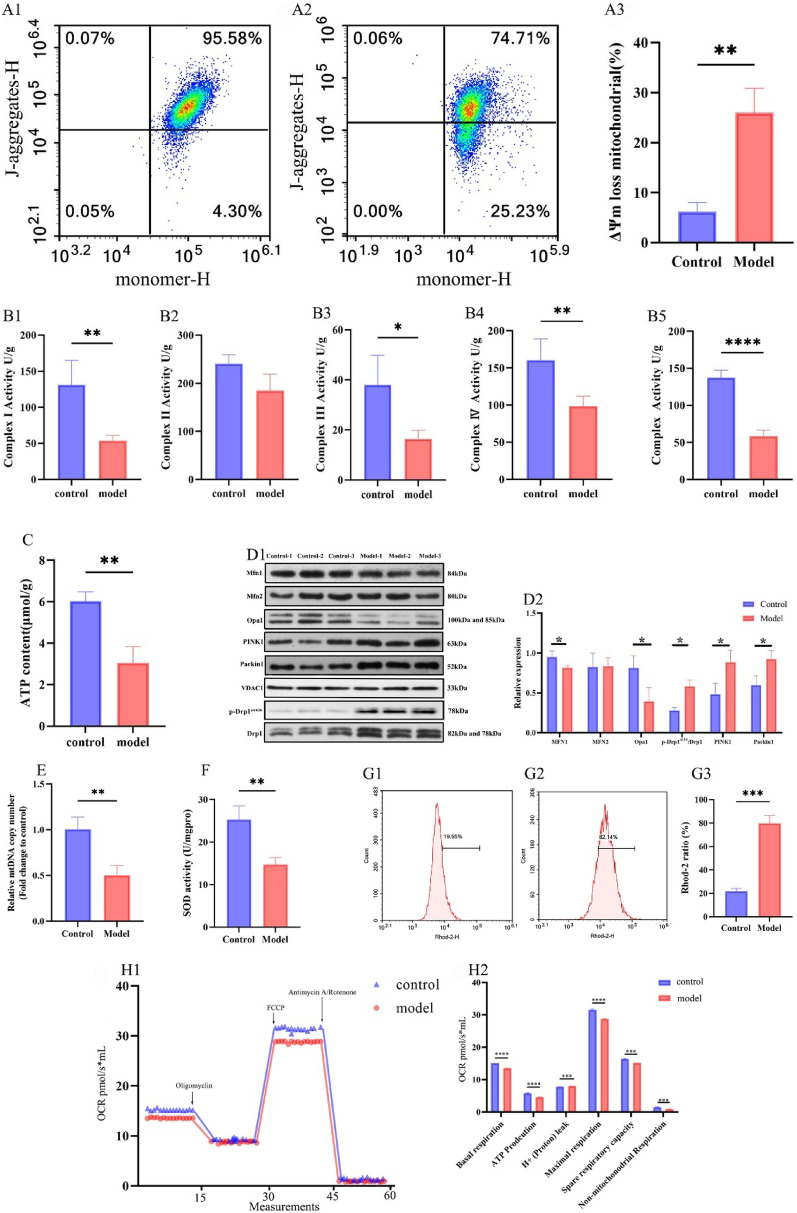
Evaluation of mitochondrial functional indicators and protein expression in the control and DCM group Note: **(A)** Mitochondrial membrane potential (ΔΨm) analysis in myocardial tissue by flow cytometry. **A1**: Control group; **A2**: DCM group; **A3**: Quantitative analysis of cells with depolarized ΔΨm, *n* = 3 per group. **(B)** Activities of respiratory chain complexes I, II, III, IV, and V in control and DCM model groups in myocardial tissue. **(C)** ATP content in myocardial tissue. **(D)** Mitochondrial dynamics and mitophagy-related protein expression in myocardial tissue. **D1**: Representative Western blot bands of Mfn1, Mfn2, Opa1, p-Drp1^Ser616^, Drp1, PINK1, and Parkin. **D2**: Quantitative analysis of protein expression levels, *n* = 3 per group. **(E)** Relative mtDNA copy number in myocardial tissue, *n* = 3 per group. **(F)** SOD activity as an oxidative stress indicator, *n* = 3 per group. **(G)** Mitochondrial Ca^2+^ levels detected by Rhod-2 fluorescence in myocardial tissue. **G1**: Control group; **G2**: DCM group; **G3**: Statistical analysis of Rhod-2 ratio (%), *n* = 3 per group. **(H)** Mitochondrial respiration assessed by OCR kinetics in H9c2 cardiomyocytes. **H1**: Real-time OCR tracing in high glucose-stimulated H9c2 cells. Arrows indicate sequential injections of oligomycin (ATP synthase inhibitor), FCCP (mitochondrial uncoupler), and antimycin A/rotenone (complex III/I inhibitors). **H2**: Quantification of the respiratory parameters derived from (H1), including basal respiration, ATP-linked respiration, proton leakage, maximal respiration, and spare respiratory capacity, calculated as described in the Methods section, *n* = 3 per group. Data are presented as mean ± standard deviation. Significance levels: *p < 0.05, **p < 0.01, ***p < 0.001, ****p < 0.0001.

Alterations in the expression of mitochondrial dynamics-related proteins can significantly impact mitochondrial morphology. Western blot analysis revealed that the expression levels of mitochondrial fusion proteins MFN1 and Opa1 were notably lower in the DCM group compared to the control group (*p* < 0.05), whereas the expression of the mitochondrial fission protein p-Drp1^Ser616^ was significantly higher (*p* < 0.05). No significant differences in the expression of the mitochondrial fusion protein MFN2 were observed between the two groups. Additionally, the expression levels of mitophagy-related proteins PINK1 and Parkin were significantly increased in the DCM group compared to the control group (*p* < 0.05) (Fig. 10D1, 2).

### Assessment of mitochondrial functional indices​

This study comprehensively assessed mitochondrial function across multiple dimensions, revealing significant impairments in mitochondrial activity in DCM. Key findings include: the proportion of mitochondria with depolarized membrane potential increased from 6.17% in the control group to 25.99% in the DCM group (*p* < 0.05) (Fig. 10A1-3). Along with the decrease in membrane potential, ATP content significantly dropped from 6.04 ± 0.44 µmol/g in the control group to 3.05 ± 0.77 µmol/g in the DCM group (*p* < 0.01) (Fig. 10C). The activities of mitochondrial respiratory chain complexes (I, II, III, IV, and V) were also evaluated to further assess respiratory function. The results demonstrated reduced activity across complexes I, III, IV, and V in the DCM group compared to controls, whereas complex II activity remained unchanged (Fig. 10B1-5). Mitochondrial integrity was further assessed by measuring the mitochondrial copy number, which was significantly lower in the DCM group compared to the control group (*p* < 0.05) (Fig. 10E). Oxidative stress in DCM mice was evaluated by measuring SOD activity in myocardial tissues. As shown in Fig. 10F, the DCM group (14.76 ± 1.63 U/mg protein) exhibited increased oxidative stress with reduced SOD activity compared to the control group (25.30 ± 3.2 U/mg protein). Furthermore, the percentage of mitochondrial Ca^2+^ was significantly elevated in the DCM group (79.77% ± 6.68%) compared to the control group (21.80% ± 2.44%) (Fig. 10G1-G3). Live-cell oxygen consumption measurements using the O2k multimode metabolic analysis system revealed that HG-treated H9c2 cells showed significant reductions in basal respiration, ATP production, maximal respiration, spare respiratory capacity, and nonmitochondrial respiration, alongside substantial increases in proton leakage (Fig. 10H1-2).

## Discussion​

Current studies on mitochondrial ultrastructural pathology predominantly rely on conventional TEM. TEM observations of biological samples require sectioning, but varying cross-sections of tissues, cells, or organelles often present different 2D perspectives, potentially leading to discrepancies between observed results and actual structures. Specific limitations include: (1) variations in the shape of the same structure across different sections; (2) the possibility of overlooking partial structures (e.g., microtubules); (3) the misinterpretation of a single structure as distinct entities; and (4) measurement errors in inter-organelle distances [14]. These challenges highlight the inherent limitations of conventional TEM in studying DCM-related ultrastructural pathology.

SEM, with its superior 3D imaging capabilities, offers a powerful tool for investigating surface topographies. Recent advancements in SEM resolution now allow backscattered electron imaging at low accelerating voltages, producing images with TEM-like contrast and resolution. By performing layered image acquisition, processing serial image stacks with specialized software, and computationally reconstructing 3D ultrastructural architectures, this technique enables comprehensive visualization of biological samples. vEM, a breakthrough technique for large-scale 3D ultrastructural analysis, was recognized as one of *Nature*’s 2023 top innovations due to its transformative scientific impact, emphasizing its irreplaceable role in elucidating disease mechanisms [15]. In this study, a preliminary 3D reconstruction of myocardial substructures was performed to provide a theoretical foundation for a deeper understanding of cardiac ultrastructure.

DCM, a diabetes-specific complication, is diagnosed by excluding coronary atherosclerosis, hypertensive heart disease, and other organic cardiac pathologies [16]. The pathogenesis of DCM involves multiple mechanisms, including hyperglycemia, dysregulated glucose/lipid metabolism, accumulation of advanced glycation end products (AGEs), inflammatory responses, and cardiomyocyte apoptosis, all of which contribute to the disease’s progression [17, 18]. Cardiomyocytes, as high-energy-demanding functional cells, rely on mitochondrial ATP production to sustain contractile function, resulting in a significantly higher mitochondrial density compared to other cell types. Mitochondrial dysfunction—manifested by altered substrate utilization, disrupted ion homeostasis, loss of mitochondrial membrane potential, excessive ROS production, and oxidative stress—directly impairs myocardial performance [19, 20]. Notably, ultrastructural abnormalities in mitochondria are critical drivers of this dysfunction.

Currently, key targets associated with the regulation of mitochondrial ultrastructure include mitochondrial dynamics and the MAM and cristae organization system. Abnormalities in both have been closely linked to the onset and progression of DCM. Mitochondrial dynamics is a highly regulated process [21, 22]. Mitochondrial fusion is mediated by mitofusin 1 (MFN1), MFN2 (outer membrane fusion), and optic atrophy 1 (OPA1) (inner membrane fusion), while fission is driven by dynamin-related protein 1 (Drp1) and fission protein 1 (Fis1) [23]. Mitochondrial fusion supports mitochondrial biogenesis and enables mtDNA exchange, preserving genomic integrity, whereas fission acts as a quality-control mechanism to segregate and autophagically remove damaged mitochondrial segments under stress [24]. Thus, exploring the role of mitochondrial dynamics in DCM is of significant importance.

Mitochondrial cristae, as invaginated structures of the inner mitochondrial membrane (IM), are the primary sites of ATP production in mitochondria. Disruption of mitochondrial cristae leads to mitochondrial dysfunction and cell death [25]. Cristae disruption is observed in various metabolic diseases [26]. Therefore, an in-depth analysis of the role of mitochondrial cristae in DCM is of critical scientific importance. This study systematically explores the relationship between mitochondrial ultrastructural changes and functional abnormalities, aiming to comprehensively unveil the 3D characteristics of mitochondrial ultrastructural pathology in DCM.

Before investigating mitochondrial ultrastructural pathology, a preliminary demonstration of myocardial tissue structures in the control group mice was performed, including T-tubules, intercalated discs, myofilaments, and blood vessels. T-tubules, unique tubular structures formed by the invagination of the cardiomyocyte sarcolemma, regulate cardiac contraction by modulating Ca^2+^ flux and action potential propagation [27]. 3D reconstruction allows for precise visualization of pathological T-tubule dilation or branching [28]. Intercalated discs, specialized junctions between adjacent cardiac muscle fibers, are essential for synchronized contractile force propagation. These structures provide mechanical connections between myofibers and mediate electrochemical communication, forming undulating architectures with repeated folding domains. The size and number of folded regions in intercalated discs can be used to assess cardiomyocyte status [29].

To evaluate 3D mitochondrial morphology, three morphometric parameters were introduced: anisotropy, flatness, and elongation. Anisotropy measures the deviation of a mitochondrion from a perfect sphere. Higher values indicate elongated or polarized shapes, while lower values (closer to 0) represent spherical uniformity. Anisotropy reflects the directional properties of mitochondria in 3D space, and its changes are often associated with mitochondrial fusion and fission [30]. Flatness quantifies the planar characteristics of mitochondria, with values approaching 0 indicating flatter structures. Changes in flatness may reflect compression in the vertical direction (e.g., swelling or membrane collapse). Elongation assesses the slenderness of mitochondria, with lower values correlating to more elongated shapes.

By analyzing these three metrics in conjunction with mitochondrial parameters such as Length 3D, Thickness 3D, Width 3D, Area, and Volume, morphological differences among the three mitochondrial subpopulations (IFM, PNM, SSM) in the myocardial tissue of the control group were observed, consistent with previously reported findings [31, 32]. Studies have demonstrated functional heterogeneity among mitochondrial subpopulations in cardiomyocytes: SSM are primarily localized near the sarcolemma and specifically generate proton motive force (PMF) in capillary-adjacent regions, whereas IFM utilize PMF to synthesize ATP for myofibril contraction. Further research has revealed that, compared to SSM, IFM exhibits significantly higher expression of OXPHOS-related proteins and superior OXPHOS capacity across diverse metabolic substrates, maintaining elevated respiratory rates under various metabolic conditions [33]. Additionally, Rajab et al. found that in early-stage diabetic mice, different mitochondrial subpopulations exhibit varying sensitivity to heterogeneity, with SSM displaying the most pronounced morphological and functional changes [11]. Given that the contractile activity of cardiomyocytes relies heavily on the structure and function of myofibrillar filaments, and since IFM are the most abundant mitochondria within the myofilaments, these mitochondria primarily specialize in highly efficient ATP production. Therefore, in assessing the differences in mitochondria between the control and DCM groups, subsequent analyses focus exclusively on the IFM.

This study explored the effects of HG exposure on mitochondrial morphology in H9c2 cardiomyocytes through in vitro experiments. Consistent with previous studies, sustained hyperglycemia induced mitochondrial shortening and fragmentation—morphological changes recognized as active regulatory mechanisms controlling ROS production and apoptosis [34, 35]. Quantitative morphometric analysis revealed that HG stimulation led to a significant reduction in mitochondrial length, while Thickness 3D and Width 3D increased significantly. In the DCM group, anisotropy decreased significantly, and elongation increased notably, collectively confirming a trend toward shorter, more rounded mitochondrial shapes. Notably, despite the fact that HG induced mitochondrial fission and fragmentation, 3D reconstruction revealed no statistically significant differences in mitochondrial Area 3D and Volume 3D between the groups, a finding that contradicts the results of Song et al. [36]. This discrepancy may be attributed to the limitations of 2D morphological assessments in previous studies, which lack the spatial resolution necessary for accurate volumetric quantification, or due to the involvement of cellular compensatory mechanisms that help maintain metabolic homeostasis following fission. However, further investigation is required to validate this hypothesis.

Numerous animal studies have demonstrated that mitochondrial dysfunction in myocardial tissue is closely associated with alterations in mitochondrial ultrastructure in DCM. For instance, Xu et al. reported significant reductions in mitochondrial cross-sectional area, perimeter, mean Feret diameter, cristae-to-mitochondrial area ratio, and crista density in DCM hearts [37]. Similarly, Chen et al. demonstrated that diabetes induces mitochondrial network fragmentation in db/db mice, leading to ferroptosis triggered by mitochondrial dysfunction [38]. Zhang et al. also observed Z-line misalignment, disorganized myofibril arrangements, mitochondrial swelling, and cristae disruption in DCM models [39]. Liu et al. showed that diabetes promotes mitochondrial fission, resulting in fragmentation and a reduced mitochondrial area, whereas paeonol—a mitochondrial fusion promoter—ameliorates DCM by restoring the fusion-fission balance [40]. Furthermore, Rajab et al. investigated mitochondrial characteristics in the hearts of GENA348 mice during the early stages of diabetes, revealing mitochondrial remodeling. Specifically, mitochondrial subpopulations exhibited altered morphology, including increased volume, irregular shape, elevated tubular projections, and higher mitochondrial density [11]. Building on these studies, the current research first quantified 2D parameters of mitochondria in fractured myocardial tissue from DCM model mice. The results confirmed previous reports, showing significant reductions in mitochondrial length, width, perimeter, and area in the DCM group. Additionally, the ShapeAP index, a metric calculated as $\frac{{{\text{(Crofton~Perimeter)2}}}}{{{\text{4}}\pi \times {\text{Area}}}}$ to assess shape complexity, anisotropy, and structural tortuosity, was introduced. This index equals 1 for a perfect circle, with higher values indicating greater topological complexity (e.g., fractal branches or elongated ellipses). The control group exhibited higher ShapeAP values, indicating more intricate mitochondrial morphologies, whereas the DCM group exhibited more isotropic, spherical structures. Using serial section-based 3D reconstruction, 3D morphological indicators were analyzed, revealing significant decreases in Width3D, Thickness3D, and Volume3D in the DCM group. These findings strongly support the pathological progression of mitochondrial fission, miniaturization, and fragmentation in DCM [41, 42].

Numerous experimental studies have demonstrated that mitochondrial dysfunction in myocardial tissue in DCM is closely associated with alterations in mitochondrial ultrastructure, with the molecular mechanisms underlying these morphological changes generally believed to be regulated by mitochondrial dynamics. High-fat and high-glucose stimulation in cardiac cells induces significant alterations in the regulatory signaling pathways governing mitochondrial dynamics. For instance, the expression levels of Opa1 and Mfn1 in cardiomyocytes are suppressed, leading to shortening of IFM [43]. Additionally, phosphorylation and activation of Drp1 trigger excessive mitochondrial fission, which contributes to cardiomyocyte injury and cardiac contractile dysfunction [44]. Transgenic mouse model studies further confirm that the deletion or downregulation of mitochondrial dynamics-related genes can induce heart failure (HF) in vivo. Disruption of MFN1- and MFN2-mediated mitochondrial fusion causes eccentric cardiac remodeling and early mortality, while Drp1 deletion accelerates DCM development [45]. Moreover, diabetes disrupts the balance of mitochondrial dynamics and mitochondrial autophagy levels in endothelial cells (ECs), contributing to cardiac microvascular injury [46]. Building on these findings, this study further measured the expression levels of proteins associated with mitochondrial dynamics and autophagy in myocardial tissue through Western blot. The results indicated a decrease in the expression levels of mitochondrial fusion proteins MFN1 and OPA1, alongside enhanced phosphorylation of the mitochondrial fission protein Drp1 and increased levels of PINK1 and Parkin, markers of mitophagy, in the DCM myocardium. These observations suggest that mitochondrial fission is significantly increased in DCM, while mitochondrial fusion is suppressed. This increased fission may serve as a cellular feedback response to high-glucose stimulation, potentially facilitating the removal of damaged mitochondria through fission followed by autophagy. The phenomenon of mitochondrial surface roughening is suggestive of structural damage to the outer mitochondrial membrane (OMM). Irregular mitochondrial surface contours were observed via two-dimensional electron microscopy in the DCM model group, while control mitochondria remained smooth and intact. Correlated observations—including Drp1-mediated mitochondrial hyperfission, collapse of the mitochondrial membrane potential (ΔΨm), and increased cardiomyocyte apoptosis—collectively indicate that surface roughening constitutes a combined morphological and functional defect under pathological stress, potentially reflecting compromised mitochondrial quality control.​.

In this study, the unchanged expression of the mitochondrial fusion protein Mfn2 suggests that Mfn1 and Mfn2 may play redundant roles in mitochondrial fusion. The reduced expression of Mfn1 and OPA1 alone may be sufficient to inhibit mitochondrial fusion. Additionally, Mfn2 appears to have nonclassical functions beyond mediating fusion, potentially operating independently of its expression level. For example, Mfn2 is localized to the ER membrane, where it forms ER-mitochondrion connections [47, 48]. Mfn2 plays a critical role in maintaining interorganellar calcium regulation and excitation-contraction-metabolic coupling in cardiomyocytes. Furthermore, upregulation of PINK1 expression not only promotes mitophagy but also enhances mitochondrial fission, working synergistically with Drp1 [49].

Mitochondrial fission is closely linked to alterations in mitochondrial function. Typically, mitochondrial fission can occur in two contexts: physiological fission, which increases mitochondrial quantity, and pathological fission, which results in mitochondrial damage or cell death. Studies have shown that decreased myocardial contractility in diabetic patients is associated with enhanced pathological mitochondrial fission [50]. Similarly, in type 2 diabetic rat models, high-glucose or high-fat stimulation induces pathological cardiac fission, manifested at the molecular level by significant downregulation of Mfn1 expression and enhanced post-translational modifications of the fission-related protein Drp1. These findings are consistent with our current observations. Excessive mitochondrial fission impairs mitochondrial function, as it leads to mitochondrial damage, disrupts OXPHOS and respiratory chain function, and ultimately induces cell death. Abnormal fission causes damage to mtDNA in daughter mitochondria, impairing their ability to express subunits of electron transport chain (ETC) complexes [51], resulting in reduced respiratory function and ATP synthesis. Additionally, uncontrolled fission significantly increases ROS production as byproducts of mitochondrial respiration [52]. This excessive fission also induces sustained opening of the mitochondrial permeability transition pore (mPTP), disrupts mitochondrial membrane potential, and increases membrane permeability, leading to cytochrome c leakage into the cytoplasm and activation of apoptosis [53]. Moreover, mitochondrial fission and ROS production are mutually reinforcing. In DCM, Yu et al. observed that H9c2 rat cardiomyocytes cultured in high-glucose medium rapidly developed mitochondrial fragmentation *via* the Drp1 signaling pathway, leading to excessive ROS production. Notably, treatment with the antioxidant TEMPOL restored normal mitochondrial morphology, suggesting that chronically elevated ROS may trigger mitochondrial fragmentation in DCM, and scavenging ROS could break this vicious cycle [35]. Additionally, excessive ROS can activate IκB kinase (IKK), which induces the phosphorylation and degradation of IκBα, thereby liberating the NF-κB dimer. The liberated NF-κB translocates into the nucleus, activating pro-inflammatory gene expression [54]. Furthermore, ROS accumulation induced by mitochondrial fission can activate the NLRP3 inflammasome, leading to cardiomyocyte pyroptosis in dilated cardiomyopathy (DCM) [55]. Furthermore, the activation of fission may also induce necroptosis [56]. Building on these findings, our study further evaluated mitochondrial integrity by measuring mtDNA copy number and analyzing ROS levels in cardiomyocytes. Consistent with previous studies, our results show that DCM disrupts mitochondrial integrity, significantly elevates ROS levels in cardiomyocytes, and is accompanied by a reduction in SOD activity.

Although mitochondria in the DCM model predominantly exhibit fragmented morphology, 3D reconstruction also revealed the presence of megamitochondria, with lengths reaching up to 6 μm. The mechanisms underlying the formation of megamitochondria and their pathological significance appear to vary depending on the tissue type. Previous studies in liver tissue have suggested that megamitochondria may form as an adaptive response to oxidative stress, reducing ROS production by suppressing ETC activity [57]. FE-SEM analyses of high-risk HF patients have identified polymorphic megamitochondria strongly associated with mtDNA abnormalities [58]. Similarly, angiotensin II (Ang II)-induced cardiac injury models have been reported to exhibit similar megamitochondria phenotypes [59]. Megamitochondria also appear in cardiomyocytes of aged mice, where they are closely linked to mitochondrial lipid metabolism dysfunction. The core mechanism involves reduced ER-mitochondrial contacts, leading to impaired lipid transport, which results in insufficient synthesis of phosphatidylethanolamine (PE) within mitochondria. This defect is further exacerbated by the downregulation of phosphatidylserine decarboxylase (PISD), the enzyme responsible for converting phosphatidylserine (PS) to PE. Reduced PISD activity leads to a significant decrease in PE levels, disrupting autophagosome membrane formation, impairing mitophagic flux, and ultimately hindering the removal of damaged mitochondria, which leads to the accumulation of dysfunctional megamitochondria [60]. In our DCM mouse model, HFD similarly induced lipid accumulation in cardiomyocytes, suggesting that DCM mice may exhibit pathological mechanisms akin to those seen in aging models. Notably, although Western blot showed upregulated expression of the PINK1/Parkin pathway in DCM mice, downstream PE-dependent LC3 lipidation remains the rate-limiting step in autophagosome membrane formation. Therefore, activation of the PINK1/Parkin pathway, which fails to effectively drive LC3-PE conjugation, cannot rectify the mitochondrial homeostasis imbalance caused by autophagic dysfunction [61]. Based on these findings, it is hypothesized that: (1) the formation of megamitochondria may represent an adaptive stress response under specific pathological conditions; and (2) the coexistence of megamitochondria with mitochondrial fragmentation reflects spatial heterogeneity in mitochondrial dynamics regulation. These findings suggest that megamitochondria formation may represent a stress-adaptive mechanism under specific pathological conditions, while their coexistence with mitochondrial fragmentation highlights spatial heterogeneity in mitochondrial dynamics regulation. However, current limitations include the unclear functional state of megamitochondria. Future studies should incorporate molecular approaches to fully elucidate their biological significance.

Mitochondria and the ER play essential roles in maintaining cellular homeostasis through critical structural and functional interactions. The key structures facilitating the exchange of information between the ER and mitochondria are known as mitochondria-associated endoplasmic reticulum membranes (MAMs) [62]. MAMs are enriched with enzymes involved in phospholipid and sphingolipid synthesis, along with various molecular chaperones. Their structural anchoring is mediated by multiple molecular bridges [63], such as the inositol 1,4,5-trisphosphate receptor (IP3R) and voltage-dependent anion-selective channel 1 (VDAC1), which form ER-mitochondrial calcium (Ca^2+^) transport channels through their interactions [64]. Moreover, the outer mitochondrial membrane protein Mfn2 anchors the ER to the mitochondrial surface by forming homomeric or heteromeric complexes with mitochondrial Mfn1/2. MAMs are critical for Ca^2+^ signaling, lipid transport, energy metabolism, and cell survival [65].

Emerging studies have emphasized the pivotal role of MAMs in the pathogenesis of DCM. For example, under diabetic conditions, inhibition of AMP-activated protein kinase (AMPK) activity promotes Fundc1-mediated MAM overformation, leading to mitochondrial Ca^2+^ overload, mitochondrial dysfunction, and DCM [66]. Furthermore, loss of the mitochondrial matrix protease LonP1 significantly inhibits MAM formation, resulting in mitochondrial fragmentation and HF related to dilated cardiomyopathy. This may be attributed to LonP1 deficiency, which promotes OPA1 processing, upregulates Drp1 expression, and suppresses MFN1 expression, ultimately enhancing mitochondrial fission [67]. These findings collectively highlight the central role of MAMs in maintaining mitochondrial homeostasis.

Building on these insights, this study systematically measured the length of MAMs and their proportional contribution to the circumference of both mitochondria and the ER in high-glucose-induced H9c2 cells and the myocardial tissues of DCM mice. The results show that both the length of MAMs and their proportion to mitochondrial circumference are significantly higher in the DCM group compared to the control group, aligning with previous findings. By combining these results with the elevated Ca^2+^ levels observed in the DCM group, it is speculated that the abnormal excessive formation of MAMs under diabetic conditions is a key factor driving Ca^2+^ overload and mitochondrial dysfunction. Inhibiting excessive MAM formation could potentially serve as a therapeutic strategy for treating DCM.

As the energy hubs of eukaryotic cells, mitochondria feature a cristae-rich inner membrane (IM) formed by characteristic invaginations, providing the structural foundation for OXPHOS and ATP synthesis. CJs are narrow channels formed by the invaginations of the IM at the necks of cristae. This structure restricts the free diffusion of substances, metabolites, and protons [68].

Under physiological conditions, crista morphology (e.g., density, continuity) undergoes adaptive remodeling in response to energy demands, while metabolic stress or apoptosis induces distinct ultrastructural alterations [69]. Adaptive crista remodeling enhances respiratory chain efficiency during metabolic challenges, whereas apoptotic crista restructuring facilitates the release of cytochrome c from the intermembrane space, activating caspase cascades and executing programmed cell death. Notably, crista abnormalities are strongly associated with various human pathologies [70, 71]. Several studies have shown that diabetes disrupts crista homeostasis. For instance, STZ-induced diabetic mouse models exhibit reduced cristae density in myocardial mitochondria [72–74]. Based on these findings, this study compared the number of cristae, crista width, crista spacing, CJ width, and crista scores. The results revealed that the DCM group exhibited fewer cristae compared to the normal control group, with increased crista width, crista spacing, and CJ width. Mechanistically, OPA1, a key regulator of IM fusion and crista architecture, is crucial for maintaining crista integrity. OPA1 localizes to the IM, stabilizing cristae junctions through coordinated membrane fusion. Hyperglycemia induces OPA1 proteolytic cleavage, resulting in decreased mitochondrial matrix electron density and vacuolar degeneration [41]. The mitochondrial contact site and cristae organizing system (MICOS) complex also plays a critical role in maintaining crista integrity [75]. MIC60 expression is reduced in the cardiac tissue of diabetic mice, while transgenic overexpression of MIC60 alleviates cardiac and mitochondrial dysfunction [76]. Moreover, competitive inhibition of MARCH5-mediated ubiquitination and degradation of MIC60 can mitigate mitochondrial cristae structural damage, dysfunction, and apoptosis in cardiomyocytes under diabetic conditions. MIC60’s shaping function is essential for CJ formation, mitochondrial membrane structure, and high-efficiency respiratory activity. In the IFM of DCM mice, MIC60 expression was significantly reduced [77]. Improvements were observed in diabetic mice with MIC60 overexpression, suggesting that high MIC60 levels exert a protective effect under diabetic conditions. Notably, the altered crista morphology in IFM under diabetic conditions was repaired in MIC60 knock-in mice, further indicating that crista remodeling plays a critical role in the onset and progression of DCM [78].

The folded architecture of mitochondrial cristae functions as a “proton trap” through spatial confinement. Protons pumped into the intermembrane space by respiratory chain complexes (I, III, IV) are sequestered within cristae lumens and directionally channeled to F_1_F_0_-ATP synthase complexes enriched at cristae tips [79–81]. This mechanism enhances ATP synthesis efficiency by optimizing the spatial distribution of the trans-inner membrane proton gradient (Δψm). Decreased ATP levels, often accompanied by mitochondrial membrane potential dissipation, indicate functional impairment, ultimately triggering apoptosis and compromising cardiac function [82, 83]. By integrating Western blot results (reduced OPA1 expression), ATP content measurements, and mitochondrial membrane potential assays, it is hypothesized that cristae structural abnormalities in DCM are linked to OPA1 downregulation. Reduced crista density may impair ATP synthesis and respiratory function through two pathways: (1) disrupted crista integrity attenuates the proton-trapping effect, leading to Δψm collapse and reduced proton gradient utilization, and (2) spatial uncoupling between respiratory chain complexes and ATP synthases disrupts OXPHOS. These findings collectively suggest that diabetes-associated crista remodeling plays a critical role in cardiomyocyte bioenergetic failure. Given that mitochondrial cristae morphology determines the activity of the oxidative respiratory chain and respiratory efficiency [84], the activity of oxidative respiratory chain complexes and OCR were further investigated.

Cardiomyocyte energy metabolism involves substrate diversity, with fatty acids serving as the primary oxidative substrate through β-oxidation to generate acetyl-CoA [85]. Glucose also produces substantial amounts of acetyl-CoA *via* glycolysis. These acetyl-CoA molecules are converted into reducing equivalents (NADH and FADH_2_) through the citric acid cycle, which subsequently participate in redox reactions *via* the ETC, composed of complexes I-IV, ultimately driving ATP synthesis *via* complex V (F_1_F_0_-ATP synthase). Measurements of ETC complex activities reflect mitochondrial respiratory function. Numerous studies have confirmed significant reductions in ETC complex activity in diabetic models [86–88]. For instance, Lashin et al. and Vazquez et al. reported approximately 30% reductions in Complexes I and II activities in STZ-induced diabetic mouse hearts, while Complexes III and IV activities remained normal [89]. In contrast, Dabkowski et al. reported marked downregulation of all ETC complexes (I-IV) in similar models [90]. In addition to redox carriers, studies also indicate reduced expression of Complex V (F_1_F_0_-ATP synthase). For example, Riu Ni et al. and Baseler et al. demonstrated significantly diminished F_1_F_0_-ATP synthase activity in STZ-treated mouse hearts [91, 92]. Our findings align with these reports, demonstrating decreased activities of Complexes I, III, IV, and V. Notably, mitochondrial OXPHOS efficiency—assessed *via* the live-cell OCR using the Oroboros O2k system—revealed significant reductions in basal respiration, ATP production, maximal respiration, spare respiratory capacity, and nonmitochondrial respiration, along with significant increases in proton leakage under high-glucose conditions. These results systematically corroborate mitochondrial bioenergetic dysfunction in DCM.

Through extensive exploration of mitochondrial ultrastructural remodeling and metabolic disturbances in DCM, coupled with the advancing understanding of mitochondrial-related mechanisms and the clarification of anti-diabetic drug mechanisms, the role of mitochondria as a therapeutic target in DCM has become increasingly prominent. Mitochondrial quality control (MQC), encompassing mitochondrial autophagy, dynamics, and biogenesis, plays a pivotal regulatory role in inflammation, oxidative stress, and cell death in cardiovascular diseases. Consequently, targeting MQC has emerged as a promising therapeutic strategy [93]. Pharmacological interventions targeting mitochondrial dynamics proteins are expected to offer novel therapeutic approaches for related diseases. For instance, compounds such as mdivi-1, P110, dynasore, and S3, which interfere with Drp1 function or inhibit MFN1/2, effectively reduce excessive mitochondrial fission, highlighting their potential therapeutic value in treating metabolic disorders [94]. The widely used anti-diabetic drug metformin has been shown to restore the balance between mitochondrial fission and fusion, activate protective mitochondrial autophagy, and restore mitochondrial metabolism and immune homeostasis, thereby improving cardiac function and offering significant cardioprotective effects [46, 95].

Furthermore, substantial evidence demonstrates that several traditional Chinese medicine (TCM) formulas and individual herbs contribute to cardiovascular protection by maintaining mitochondrial homeostasis. For example, Tongyang Huoxue decoction inhibits excessive mitochondrial fission by maintaining β-tubulin levels and activating SIRT1, while also stimulating mitochondrial autophagy and increasing mitochondrial membrane potential, ultimately preserving mitochondrial function and protecting sinoatrial node cells (SNCs) [96]. Zishen Tongyang Huoxue decoction (TYHX) alleviates ischemia/reperfusion injury in SNCs by regulating MQC through the VDAC1–β-tubulin signaling axis, altering VDAC1 permeability, modulating β-tubulin-mediated MQC, activating FUNDC1-mediated mitophagy, inhibiting excessive mitochondrial fission and overactive unfolded protein response (UPRmt), and restoring mitochondrial dynamics [97]. Another TCM formula, Zishenhuoxue decoction (ZSHX), improves cardiac function and reduces myocardial ischemic injury by regulating myocardial mitochondrial calcium homeostasis and restoring MQC balance *via* the TMBIM6-VDAC1 axis [98]. Quercetin helps maintain MQC in cardiomyocytes under ischemia-reperfusion stress by promoting the interaction between DNA-PKcs and SIRT5, which coordinately regulates mitochondrial autophagy and the unfolded protein response [99]. Ginsenoside Rb1 alleviates HF by targeting the DUSP-1-TMBIM6-VDAC1 signaling axis, coordinating MQC regulation, inhibiting NLRP3 inflammasome-mediated inflammation and pyroptosis, modulating gut microbiota composition, and establishing a protective network, ultimately improving myocardial injury and cardiac function in HF mice [100].

Limitations:

This study systematically elucidated the synergistic pathological mechanisms of mitochondrial ultrastructural changes and functional disorders in DCM through the integration of 3D visualization technology and molecular biology approaches. Despite these advancements, several limitations and areas requiring further exploration remain. These include:

(1) Technical limitations: The study utilized serial sectioning 3D reconstruction technology to analyze mitochondrial ultrastructure. While this method avoids the high costs associated with vEM, it presents challenges such as difficulties in slice collection, complex post-processing of images, and limited Z-axis resolution. In contrast, advanced techniques such as focused ion beam scanning electron microscopy (FIB-SEM) and TEM electron tomography (Electron Tomography) offer more precise ultrastructural parsing, while 3-View SEM and automated tape-collecting ultramicrotome (ATUMtome) are better suited for acquiring larger-scale myocardial ultrastructures. Thus, future 3D structural studies should carefully select technical approaches based on experimental conditions and research objectives. (2) Mechanistic limitations: This study predominantly employed 3D electron microscopy to observe and quantitatively analyze mitochondrial morphology in myocardial tissues of DCM model mice, alongside evaluating the expression of mitochondrial dynamics and autophagy-related proteins, as well as functional indicators such as mitochondrial membrane potential and OCR. However, the molecular regulatory mechanisms underlying these observations were not thoroughly explored. Subsequent studies should employ gene knockout/knockdown or overexpression techniques to target specific genes and clarify their molecular mechanisms in greater detail. (3) Dynamic research limitations: While this study focused on static pathological features of mitochondrial ultrastructural remodeling and functional impairment in DCM, the dynamic evolutionary process of mitochondrial remodeling and functional decline remains poorly understood. Future research utilizing live-cell dynamic imaging technologies, such as fluorescently labeled mitochondrial tracking, could provide insights into the temporal patterns of mitochondrial morphology-function changes and their causal relationship with cardiac dysfunction. (4) Molecular basis of mitochondrial heterogeneity: Although numerous studies have documented the presence of distinct mitochondrial subpopulations in cardiomyocytes, exhibiting significant morphological and functional heterogeneity, the molecular mechanisms driving this heterogeneity remain unclear. Future research could leverage spatial transcriptomics or single-cell mitochondrial proteomics to profile subpopulation-specific protein expression and investigate how this heterogeneity correlates with regional regulation of cardiac energy metabolism. (5) Clinical translation and intervention limitations: Research on the ultrastructural pathology of myocardial mitochondria aims to provide new insights into the pathogenesis of DCM, laying a foundation for subsequent clinical applications. Building upon the evidence linking ultrastructure to function, future studies could incorporate intervention strategies targeting mitochondrial dynamics (e.g., Drp1 inhibitors) or mitochondrial cristae stability (e.g., MIC60 inhibitors) to explore novel therapeutic approaches. Additionally, evaluating the pharmacological effects of TCM formulas or individual herbs in regulating mitochondrial function and improving DCM could offer valuable therapeutic avenues.

## Conclusion

This study elucidates the synergistic pathogenic mechanisms underlying mitochondrial ultrastructural abnormalities and bioenergetic dysfunction in DCM through a multidimensional technical approach. The key findings are as follows: (1) 3D ultrastructural remodeling: Mitochondria in DCM exhibit pronounced fragmentation and cristae disruption, with 3D visualization techniques enabling the identification of megamitochondria—a pathological phenomenon not detectable by conventional 2D electron microscopy. (2) Molecular dynamics imbalance: Coordinated downregulation of mitochondrial fusion proteins (Mfn1, OPA1) and aberrant activation of the fission protein p-Drp1^ser616^ drive ultrastructural remodeling. (3) Bioenergetic failure: In DCM, myocardial tissue enters a vicious cycle marked by mitochondrial membrane potential dissipation, impaired activity of respiratory chain complexes (I, III, IV, and V), and deficits in ATP synthesis. This cascade ultimately contributes to myocardial contractile dysfunction. Additionally, a reduction in mtDNA copy number, decreased SOD activity, and elevated mitochondrial Ca^2+^ levels are observed. High glucose stimulation further leads to a decreased OCR in H9c2 cells. By integrating 3D morphometric analysis with functional profiling, this study ​uncovers​ concurrent dysregulation in structural organization, molecular signaling, and bioenergetic function, ​thereby establishing​ a multidimensional framework for DCM pathogenesis, offering a novel theoretical framework for understanding DCM pathogenesis and laying the scientific groundwork for the development of mitochondrion-targeted therapeutic strategies.

## Supplementary Information

Below is the link to the electronic supplementary material.

Supplementary Material 1

## Data Availability

No datasets were generated or analysed during the current study.

## Abbreviations

DCM: Diabetic cardiomyopathy
SEM: Scanning electron microscopy
TEM: Transmission electron microscopy
vEM: Volume electron microscopy
SBF‒SEM: Serial block-face scanning electron microscopy
HFD: High-fat diet
STZ: Streptozotocin
LVEF: Left ventricular ejection fraction
LVFS: Left ventricular fractional shortening
LVIDd: Left ventricular internal diameter at end-diastole
IVSd: Interventricular septal thickness at end-diastole
LVPWd: Left ventricular posterior wall thickness at end-diastole
HE: Hematoxylin-Eosin
DMEM: Dulbecco’s modified eagle’s medium
FBS: Fetal bovine serum
HG: High glucose
PB: Phosphate buffer
DMSO: Dimethyl sulfoxide
OsO₄: Osmium tetroxide
RT: Room temperature
SDS‒PAGE: Sodium dodecyl sulfate‒polyacrylamide gel electrophoresis
PVDF: Polyvinylidene fluoride
BCA: Bicinchoninic acid assay
ECL: Enhanced Chemiluminescence
JC-1: 5,5′,6,6′-Tetrachloro-1,1′,3,3′-tetraethylbenzimidazolocarbocyanine Iodide
FCCP: Carbonyl Cyanide-4-(Trifluoromethoxy)Phenylhydrazone
OCR: Oxygen consumption rate
SSM: Subsarcolemmal mitochondria
IFM: Interfibrillar mitochondria
PNM: Perinuclear mitochondria
MFN1: Mitofusin 1
OPA1: Optic atrophy 1
DRP1: Dynamin-related protein 1
PINK1: PTEN-induced putative kinase 1
PARKIN: Parkin RBR E3 Ubiquitin protein ligase
ATP: Adenosine triphosphate
ΔΨm: Mitochondrial membrane potential
ETC: Electron transport chain
ROS: Reactive oxygen species
OXPHOS: Oxidative phosphorylation
SR: Sarcoplasmic reticulum
ER: Endoplasmic reticulum
mtDNA: Mitochondrial DNA
IM: Inner mitochondrial membrane
AGEs: Advanced glycation end products
FIS1: Fission protein 1
TUNEL: Terminal deoxynucleotidyl transferase dUTP nick end labeling
VDAC: Voltage-dependent anion channel
mPTP: Mitochondrial permeability transition pore
