# Supplementary figures and images for: Mitochondrial ultrastructural pathology in diabetic cardiomyopathy: integrated analysis via scanning electron microscopy and 3D visualization imaging
Source: Cardiovasc Diabetol. 2025 Aug 13;24:331. doi: 10.1186/s12933-025-02884-5 (PMC12345013; doi:10.1186/s12933-025-02884-5)

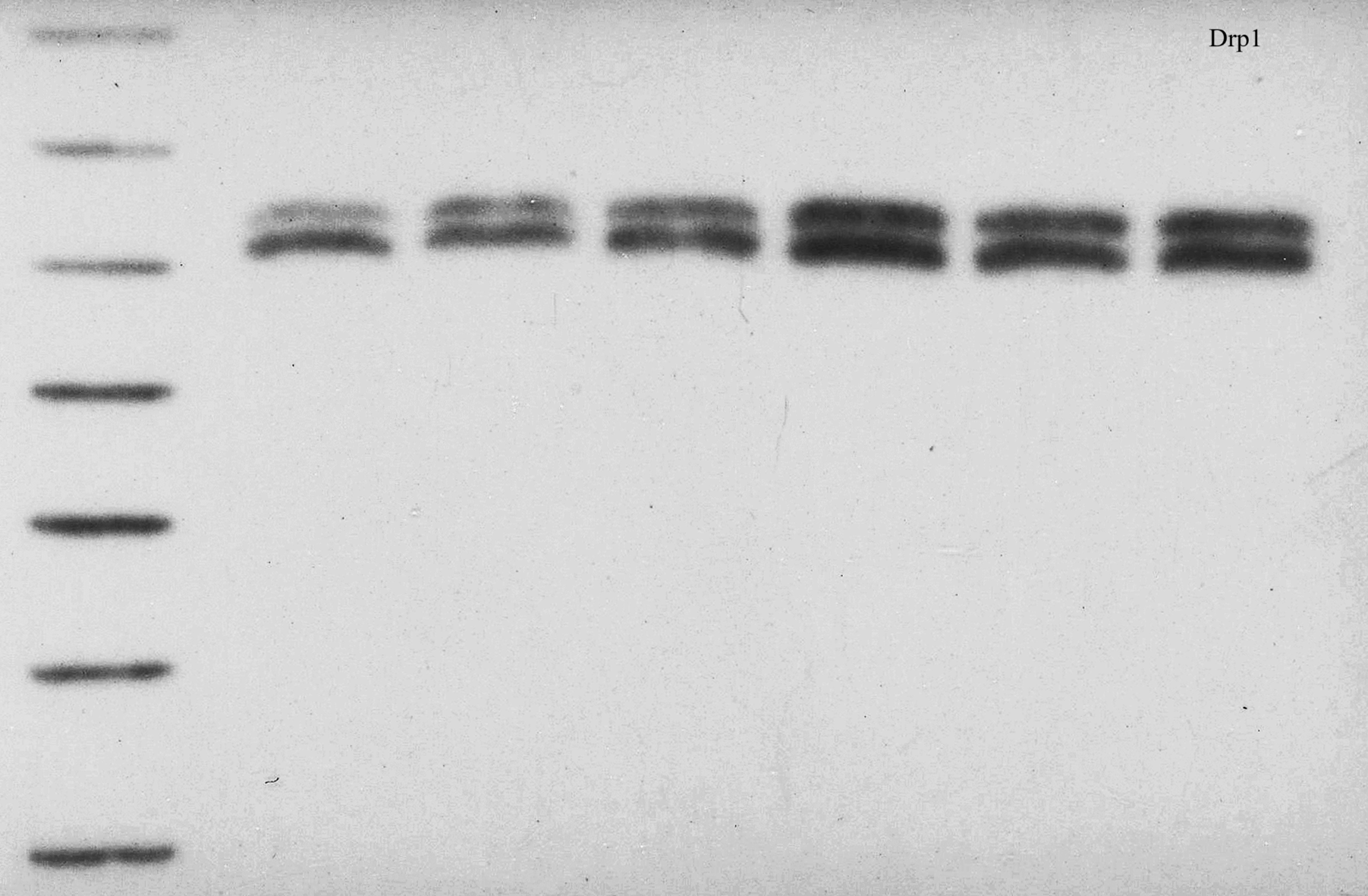

Supplement: Supplementary file 1 — Supplementary Material 1 [file 12933_2025_2884_MOESM1_ESM.zip › supplement/Gels and Blots image(s)/Drp1.tif]

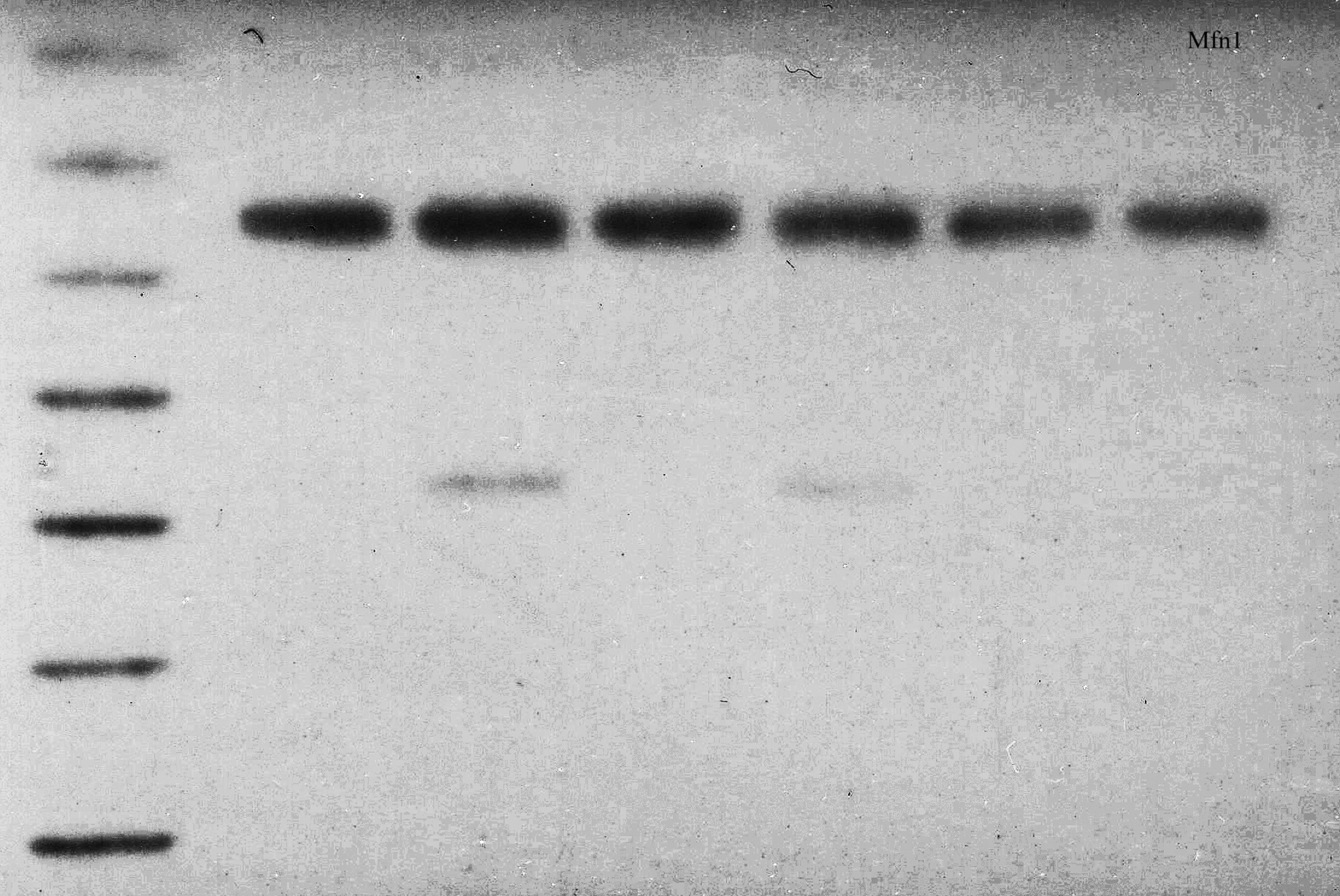

Supplement: Supplementary file 1 — Supplementary Material 1 [file 12933_2025_2884_MOESM1_ESM.zip › supplement/Gels and Blots image(s)/Mfn1.tif]

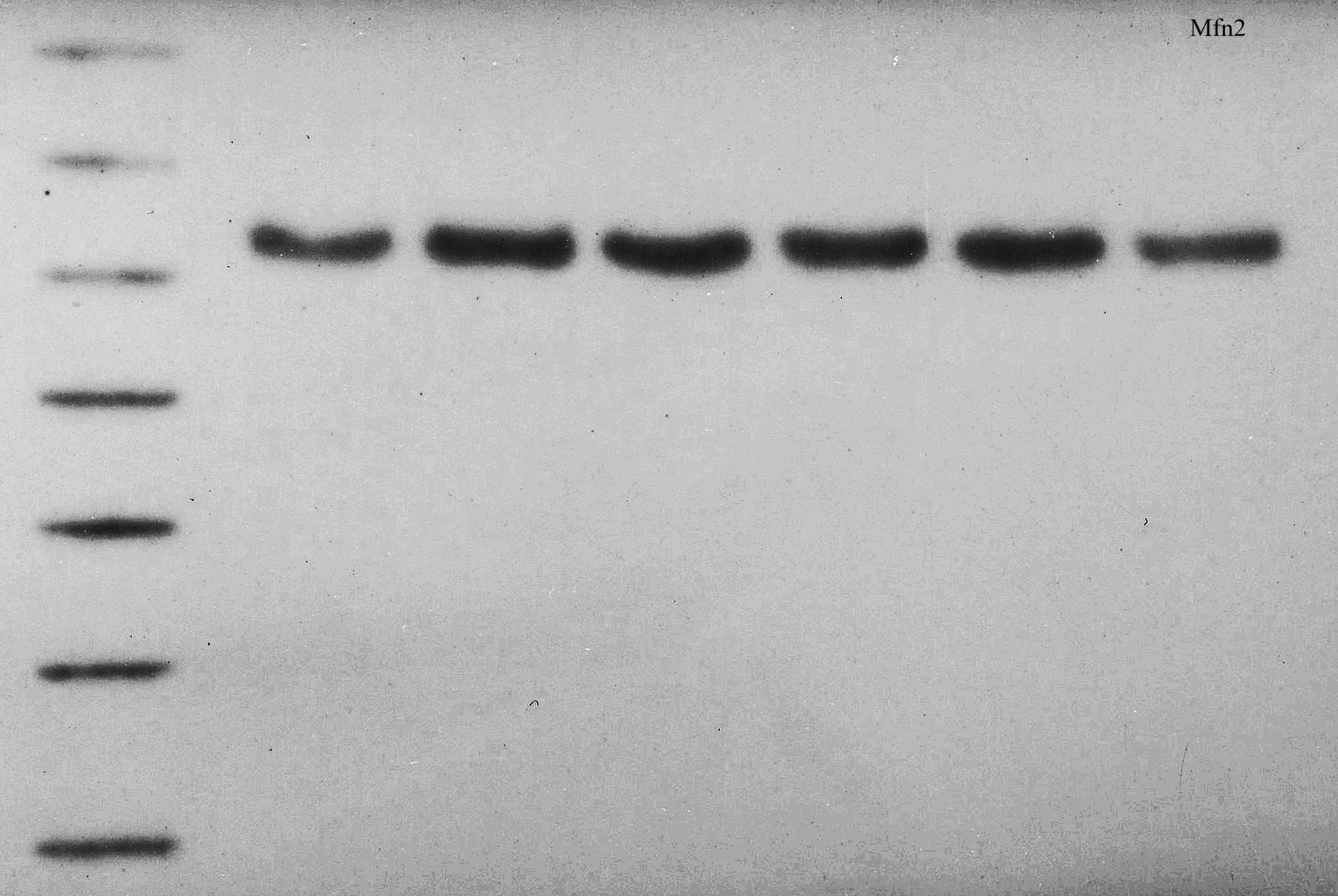

Supplement: Supplementary file 1 — Supplementary Material 1 [file 12933_2025_2884_MOESM1_ESM.zip › supplement/Gels and Blots image(s)/Mfn2.tif]

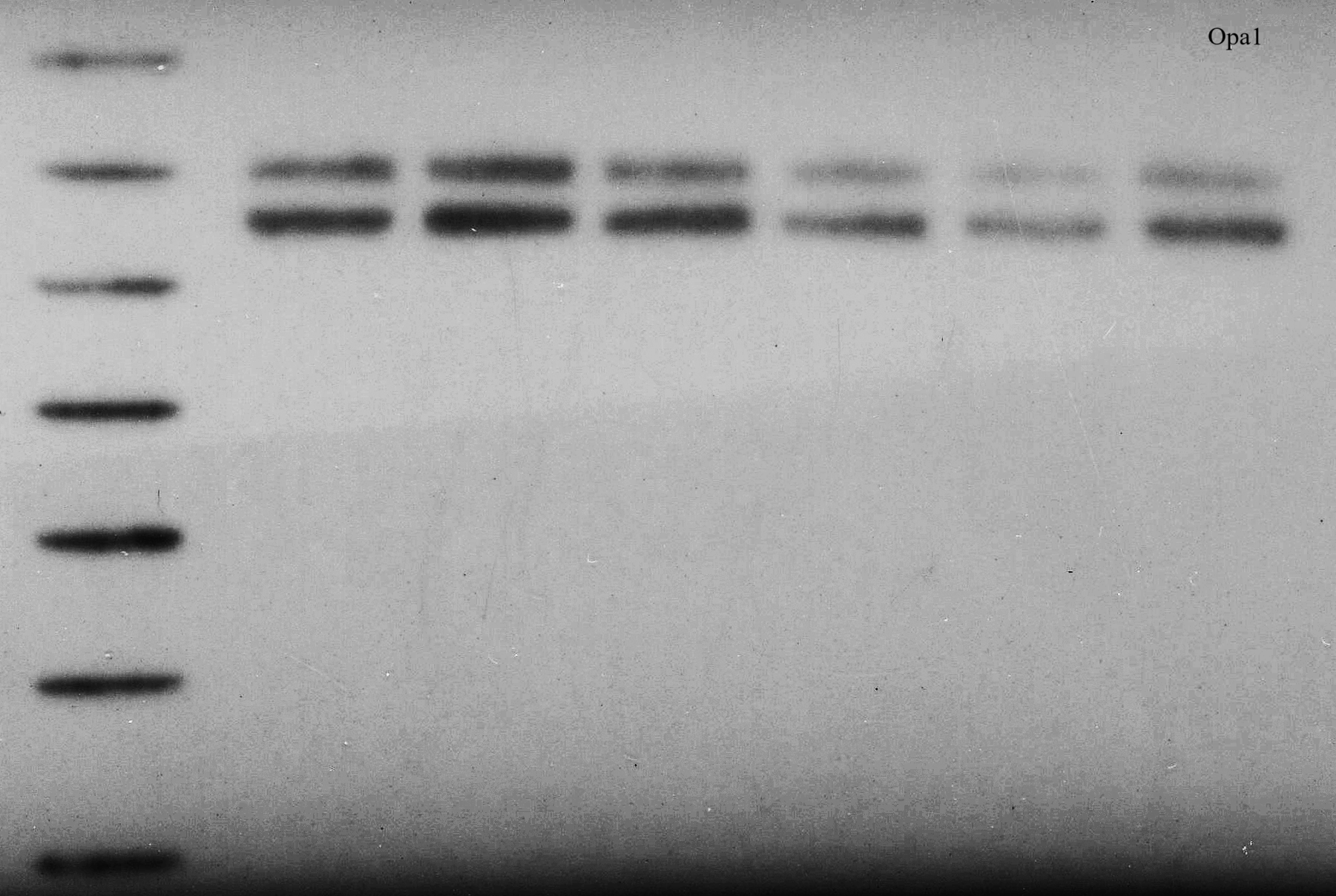

Supplement: Supplementary file 1 — Supplementary Material 1 [file 12933_2025_2884_MOESM1_ESM.zip › supplement/Gels and Blots image(s)/Opa1.tif]

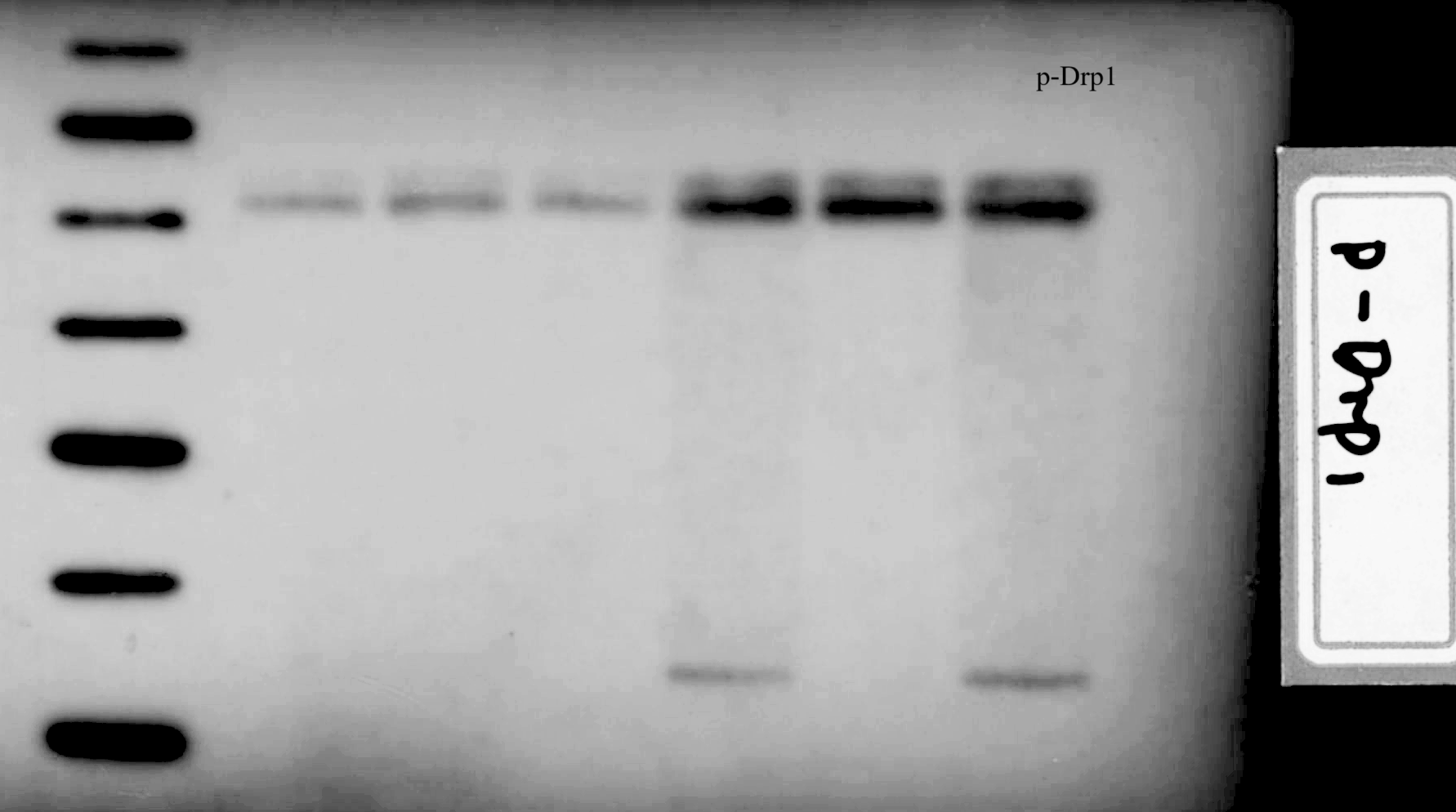

Supplement: Supplementary file 1 — Supplementary Material 1 [file 12933_2025_2884_MOESM1_ESM.zip › supplement/Gels and Blots image(s)/p-Drp1.tif]

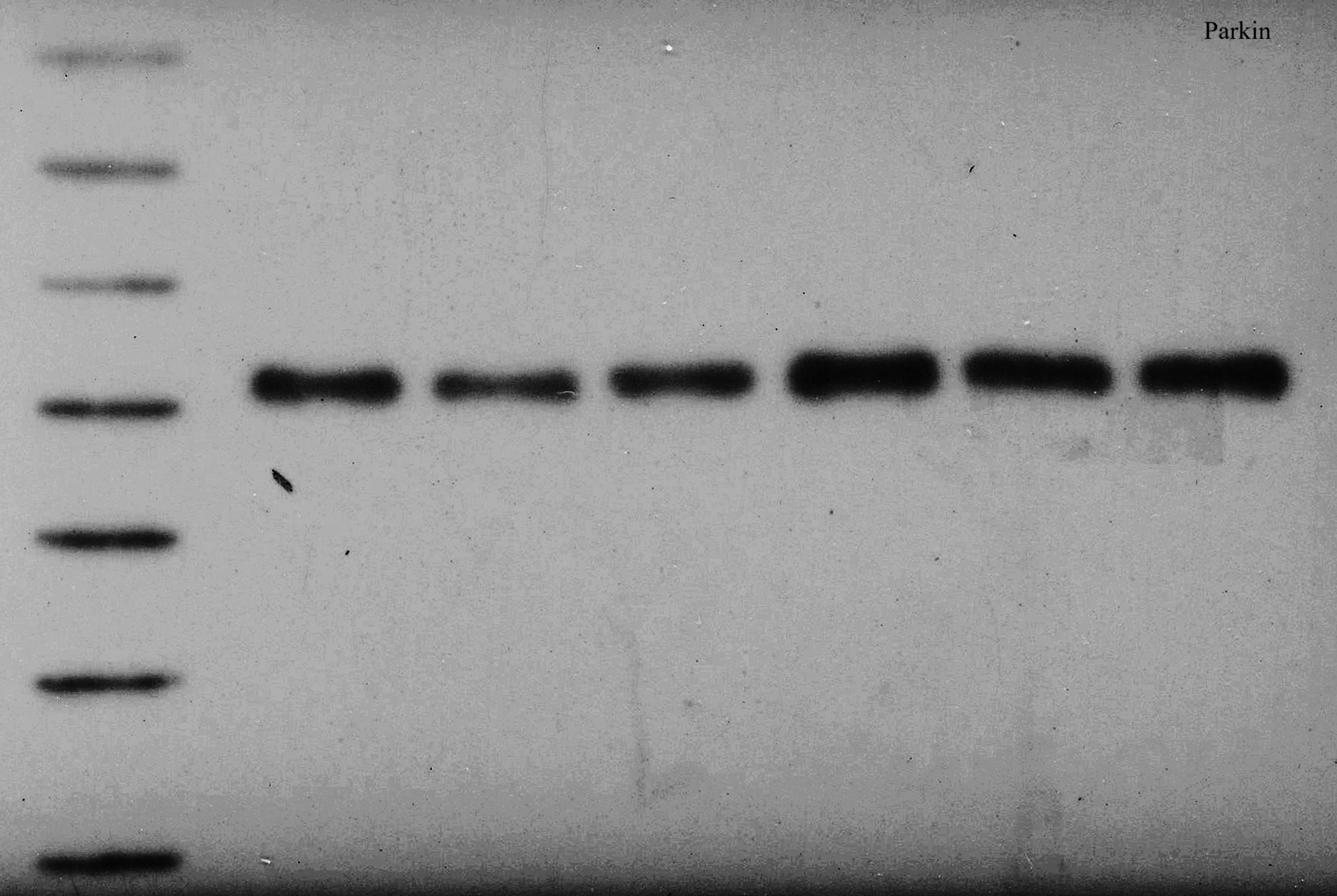

Supplement: Supplementary file 1 — Supplementary Material 1 [file 12933_2025_2884_MOESM1_ESM.zip › supplement/Gels and Blots image(s)/Parkin.tif]

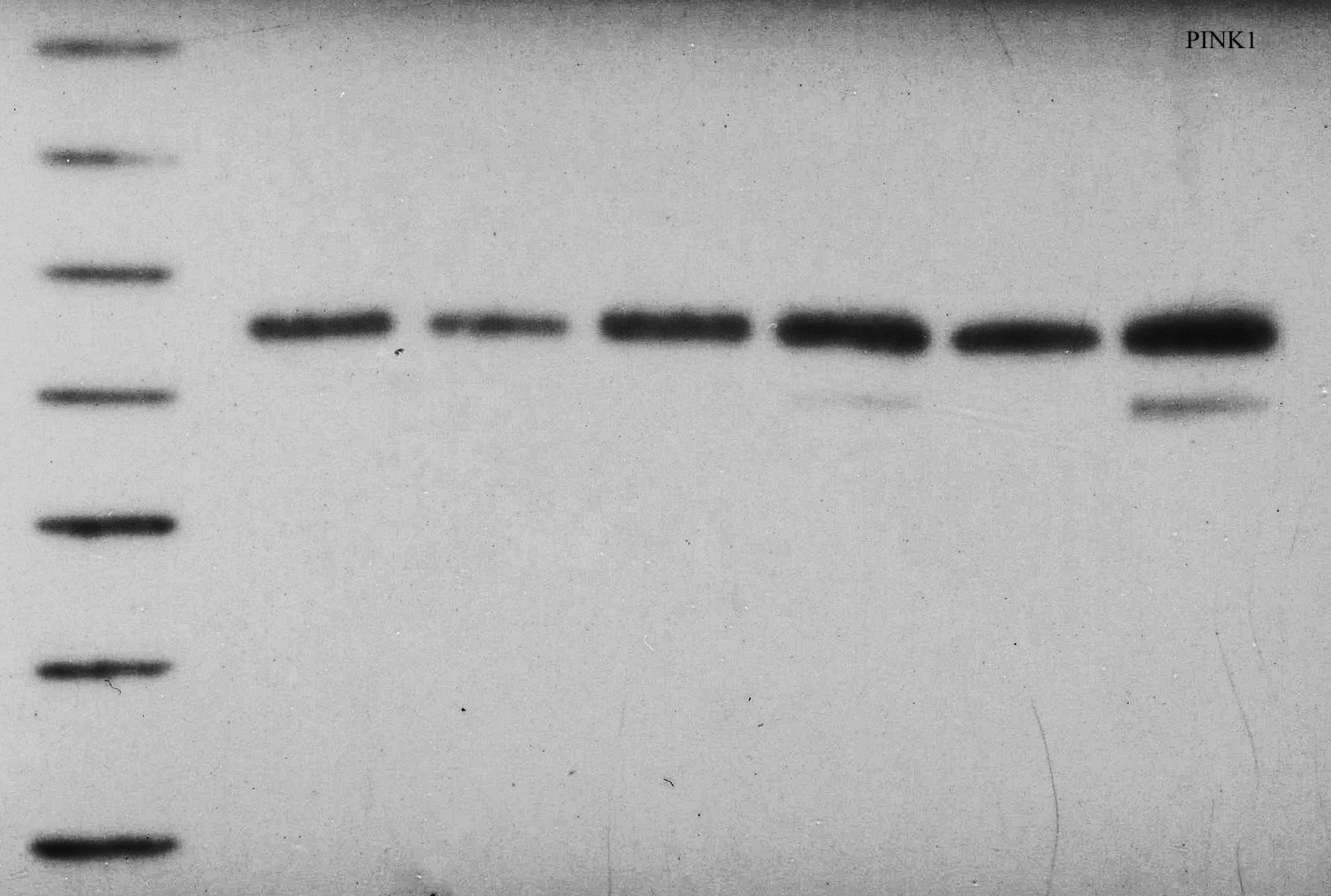

Supplement: Supplementary file 1 — Supplementary Material 1 [file 12933_2025_2884_MOESM1_ESM.zip › supplement/Gels and Blots image(s)/PINK1.tif]

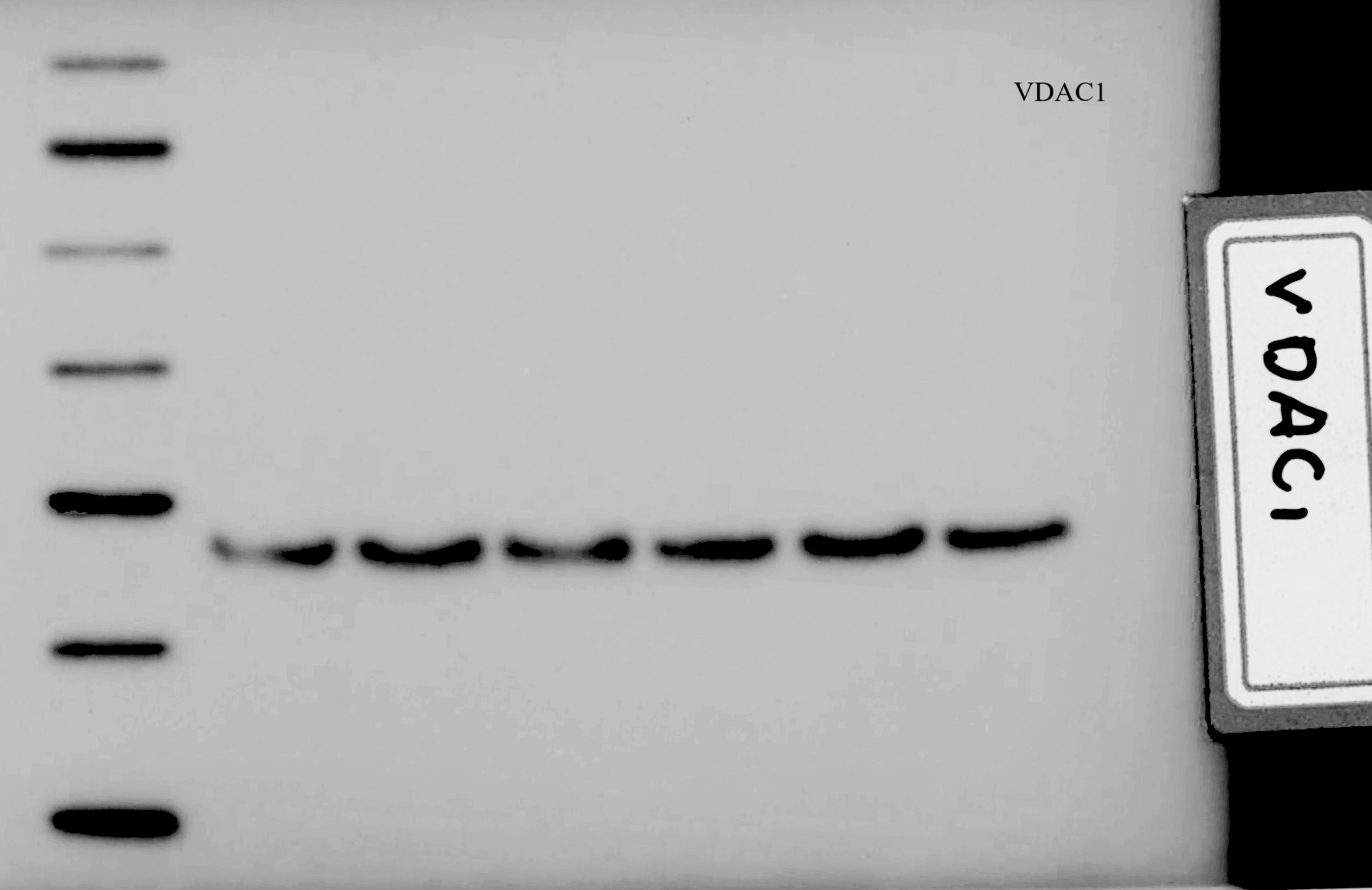

Supplement: Supplementary file 1 — Supplementary Material 1 [file 12933_2025_2884_MOESM1_ESM.zip › supplement/Gels and Blots image(s)/VDAC1.tif]
